# Comparative Genomics of Synechococcus elongatus Explains the Phenotypic Diversity of the Strains

**DOI:** 10.1128/mbio.00862-22

**Published:** 2022-04-27

**Authors:** Marie Adomako, Dustin Ernst, Ryan Simkovsky, Yi-Yun Chao, Jingtong Wang, Mingxu Fang, Christiane Bouchier, Rocio Lopez-Igual, Didier Mazel, Muriel Gugger, Susan S. Golden

**Affiliations:** a Division of Biological Sciences, University of California, San Diego, La Jolla, California, USA; b Center for Circadian Biology, University of California, San Diego, La Jolla, California, USA; c Institut Pasteur, Université de Paris, Genomic Platform, Paris, France; d Institut Pasteur, Université de Paris, Unité de Plasticité du Génome Bactérien, et CNRS, Paris, France; e Institut Pasteur, Université de Paris, Collection of Cyanobacteria, Paris, France; f Instituto de Bioquímica Vegetal y Fotosíntesis, CSIC and Universidad de Sevilla, Seville, Spain; University of Washington

**Keywords:** *Synechococcus*, biofilm, circadian rhythms, comparative genomics, cyanobacteria, phototaxis

## Abstract

Strains of the freshwater cyanobacterium Synechococcus elongatus were first isolated approximately 60 years ago, and PCC 7942 is well established as a model for photosynthesis, circadian biology, and biotechnology research. The recent isolation of UTEX 3055 and subsequent discoveries in biofilm and phototaxis phenotypes suggest that lab strains of S. elongatus are highly domesticated. We performed a comprehensive genome comparison among the available genomes of S. elongatus and sequenced two additional laboratory strains to trace the loss of native phenotypes from the standard lab strains and determine the genetic basis of useful phenotypes. The genome comparison analysis provides a pangenome description of S. elongatus, as well as correction of extensive errors in the published sequence for the type strain PCC 6301. The comparison of gene sets and single nucleotide polymorphisms (SNPs) among strains clarifies strain isolation histories and, together with large-scale genome differences, supports a hypothesis of laboratory domestication. Prophage genes in laboratory strains, but not UTEX 3055, affect pigmentation, while unique genes in UTEX 3055 are necessary for phototaxis. The genomic differences identified in this study include previously reported SNPs that are, in reality, sequencing errors, as well as SNPs and genome differences that have phenotypic consequences. One SNP in the circadian response regulator *rpaA* that has caused confusion is clarified here as belonging to an aberrant clone of PCC 7942, used for the published genome sequence, that has confounded the interpretation of circadian fitness research.

## INTRODUCTION

Cyanobacteria are important on a global scale as widespread primary producers in environments as diverse as the world’s oceans, rivers, freshwater lakes, and deserts (1–3). In addition to their roles in natural environments, cyanobacteria have attracted interest for their use as biotechnology production platforms (4). Synechococcus elongatus PCC 7942 is a well-studied freshwater cyanobacterium long established as a cyanobacterial model organism used for research in prokaryotic photosynthesis and circadian rhythms, as well as one of a few cyanobacterial model strains adopted for biotechnology purposes (5–7). Its model status accrues from its facile genetic manipulation based on natural transformability and robust homologous recombination machinery (8), along with a small genome, planktonic growth habit, and formation of distinct colonies on plates. In addition to PCC 7942, there are four other strains of S. elongatus with nearly identical genomes that did not reach the same status, due to either their loss of natural competence or historical quirks of fate (9).

Recent discoveries raised the likelihood that lab strains of S. elongatus are highly domesticated. For example, under laboratory culturing conditions, PCC 7942 exhibits a persistent suspended planktonic phenotype, even in the absence of agitation or bubbling, with no evidence of biofilm formation on the culture vessel. Schatz et al. identified and characterized a biofilming mutant of PCC 7942 (10). Studies using conditioned media showed that the wild-type (WT) lab strain secretes an unknown repressor of biofilm formation, supporting a model of constitutive repression of the biofilm genetic program in PCC 7942. This model, coupled with a 40-year history of lab adaptation for the strain that may have favored planktonic growth, led to a hypothesis that an environmental isolate of S. elongatus would readily form biofilms.

As a test of this hypothesis, S. elongatus UTEX 3055 was isolated from Waller Creek, Texas, United States, in 2014 and was found to share 98.5% nucleotide identity with PCC 7942. Although clearly the same species as PCC 7942, the genome of UTEX 3055 is notably distinct from that of PCC 7942. Moreover, UTEX 3055 forms biofilms in laboratory conditions and is phototactic, with an unusual photoreceptor that controls bidirectional phototaxis (11). Although PCC 7942 is not phototactic, genetic transplantation of the genes for the photoreceptor and other components of the phototaxis pathway from PCC 7942 to UTEX 3055 showed that the photoreceptor genes of PCC 7942 are functional, and phototaxis may be an intrinsic property of PCC 7942 that was lost during laboratory propagation (11). We hypothesized the loss of phenotypes like biofilm formation and phototaxis from the standard lab strains of S. elongatus through domestication during laboratory cultivation might be traceable using comparative genomics.

The isolation history of S. elongatus strains is the context for understanding the connection between their phenotypes and genotypes. The legacy strains of S. elongatus include the earliest isolations from freshwater sources in Texas (PCC 6301; alias, UTEX 625) and southern California (PCC 6311) (12) and strains later isolated from freshwater near San Francisco, California (9). As the earliest isolate and entry in cyanobacterial culture collections, PCC 6301 became the type strain for S. elongatus. One of the San Francisco strains was found to be highly transformable and genetically very similar to another transformable strain of unknown isolation history in a collection in Russia (13, 14), and these strains were deposited in the Pasteur Culture Collection as PCC 7942 and PCC 7943, respectively. PCC 6311 and PCC 7943 were sequenced for this study. The last legacy strain, UTEX 2973, was isolated recently from a frozen archive of UTEX 625 (PCC 6301) (15). In 2015, UTEX 3055 was isolated from Waller Creek, Texas, about 60 years after PCC 6301 was sampled from the same source (11).

We undertook a comprehensive genome comparison among UTEX 3055 and the previously characterized S. elongatus isolates PCC 6301, PCC 6311, PCC 7942, PCC 7943, and UTEX 2973, here referred to as “legacy strains.” The first results of this analysis are sequence and annotation refinement through resequencing of the type strain PCC 6301 and sequencing of PCC 6311 and PCC 7943, as well as the creation of a curated pangenome annotation for all S. elongatus strains. Examination of the genome differences at successively narrowing scales reveals large genome regions that control pigmentation phenotypes, a putative operon of UTEX 3055 necessary for phototaxis, patterns of single nucleotide polymorphisms (SNPs) in legacy strains that led to a reevaluation of the relationships among PCC 6301, PCC 7942, and UTEX 2973, and an explanation of a perplexing SNP in *rpaA*, the master regulator output of the circadian clock, which has previously caused confusion in the literature.

## RESULTS AND DISCUSSION

### A pangenome analysis approach refines genome sequences and annotations.

A pangenome compilation strategy using whole-genome alignments and ortholog comparisons was adopted to facilitate comparisons among S. elongatus strains since some strains have DNA segments that are unique or absent relative to others. The pangenome of S. elongatus contains 3,079 genes, with a shared core genome of 2,632 genes. There is high sequence conservation among core genome genes, and yet, ~15% of the annotations varied among genomes (Data Set S3 in the supplemental material). These annotation variations were adjusted using available published transcriptomics (16, 17), gene essentiality (18), and transcriptome sequencing (RNA-Seq) (16) data for PCC 7942 to create a universal S. elongatus pangenome annotation. The pangenome annotation adjusts 178 gene annotations, removes pseudogenes and hypothetical annotations that lack transcriptional evidence, and adds noncoding RNAs with transcriptional and essentiality evidence in PCC 7942. The pangenome annotations and associated metadata are available as Data Set S1.

10.1128/mbio.00862-22.1 DATA SET S1 Pangenome annotations and associated metadata for S. elongatus strains used in this study. Download Data Set S1, XLSX file, 1.3 MB. Copyright © 2022 Adomako et al. 2022 Adomako et al. https://creativecommons.org/licenses/by/4.0/ This content is distributed under the terms of the Creative Commons Attribution 4.0 International license.

10.1128/mbio.00862-22.3 DATA SET S3 Additional gene sets and comparisons used in this study. Download Data Set S3, XLSX file, 0.03 MB. Copyright © 2022 Adomako et al. 2022 Adomako et al. https://creativecommons.org/licenses/by/4.0/ This content is distributed under the terms of the Creative Commons Attribution 4.0 International license.

The type strain PCC 6301 was one of the first cyanobacterial genomes sequenced before the advent of next-generation sequencing (19). When the published sequence of PCC 6301 is compared with the other legacy strains, there appear to be more than 1,000 SNPs and insertion-deletion events (indels) in PCC 6301 (15). However, close examination showed that many apparent SNPs in PCC 6301 result in frameshift or nonsense mutations in genes that are essential for viability in PCC 7942 (18). A sample of PCC 6301 archived cryogenically in 1988 in the Golden lab was resequenced, and this updated sequence contains none of the previously observed SNPs in essential genes. This new sequence (GenBank accession nos. CP085785 to CP085787) was used in the subsequent analyses in this paper and is recommended for any future genomic comparison analysis that uses PCC 6301 as the type strain of S. elongatus.

**FIG 1:**
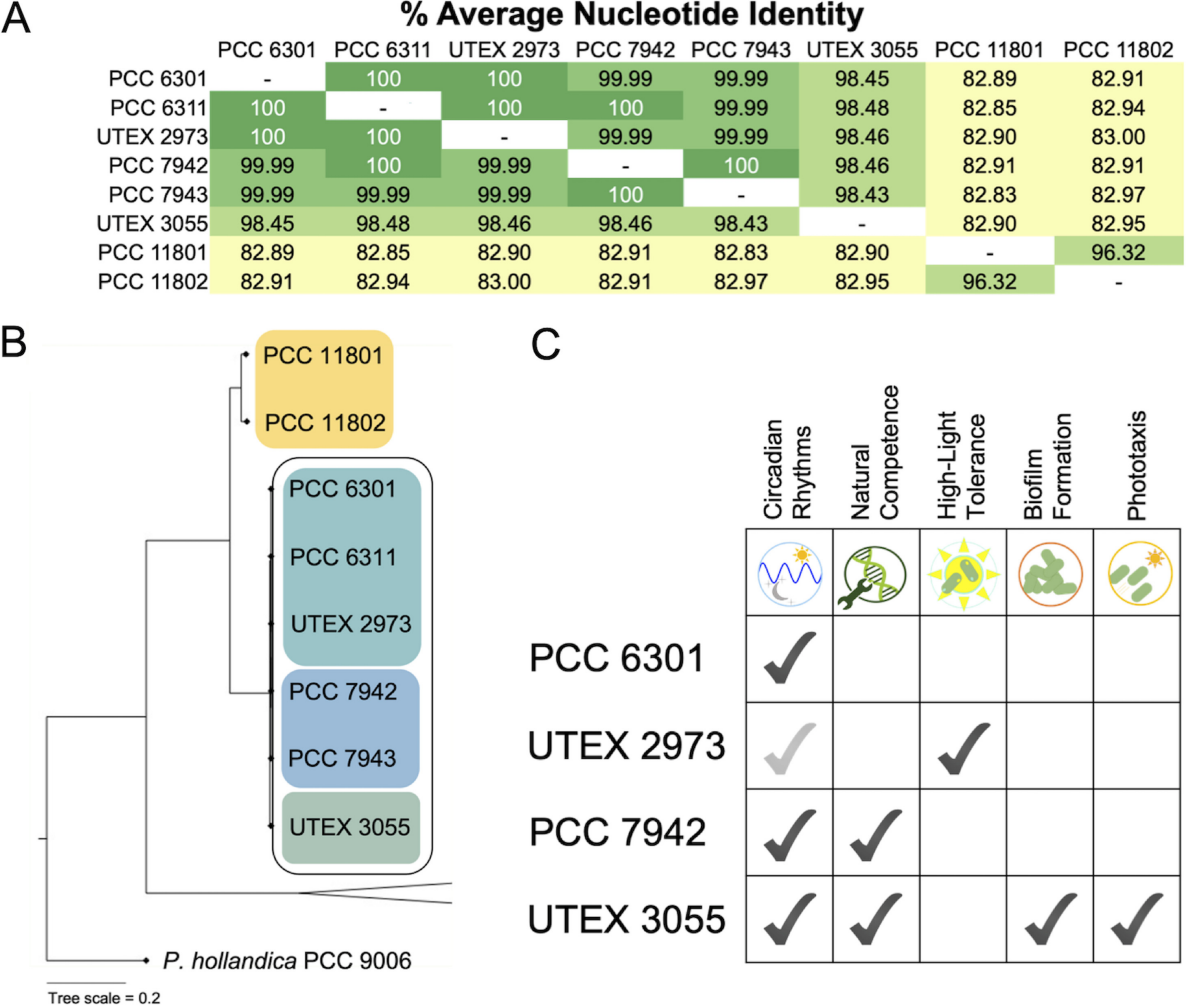
The legacy strains and UTEX 3055 share high average nucleotide identity (A), cluster in a monophyletic group separately from other *Prochlorococcus* and *Synechococcus* species (see expanded phylogenetic tree in Fig. S1 in the supplemental material) (B), and have unique combinations of phenotypes (gray checkmark represents presumed circadian rhythms in UTEX 2973; the strain has never been explicitly tested for rhythmic gene expression) (C).

S. elongatus strains share an average nucleotide identity of 98.5%, and yet, they have distinct phenotypes in natural competence, light tolerance, phototaxis, and biofilm formation (Fig. 1A and C). The legacy strains share an even higher average nucleotide identity of 99.9%, and yet, previous genome comparison studies have found SNPs that contribute to the high-light-tolerance phenotype of UTEX 2973 (15, 20, 21) and the loss of natural competence in PCC 6301 and UTEX 2973 (22). There are reports of transformation of PCC 6301 in the early literature (23–25) at the same time as reports of the superior transformability of PCC 7942 (26). PCC 6301 contains an SNP resulting in a frameshift mutation that inactivates the type IV pilus component *pilN* necessary for transformation, a mutation shared with UTEX 2973 and PCC 6311. Considering the tens of genes required for natural competence in S. elongatus (22, 27), it is unsurprising that natural competence would be lost in laboratory strains that are not actively propagated for the trait and that this difference in transformability paved the way for PCC 7942 to enter labs around the world as a genetically tractable cyanobacterial model.

10.1128/mbio.00862-22.4 FIG S1 Phylogenetic tree of S. elongatus strains with representative species from the *Prochlorococcus* and *Synechococcus* clade. Download FIG S1, PDF file, 0.04 MB. Copyright © 2022 Adomako et al. 2022 Adomako et al. https://creativecommons.org/licenses/by/4.0/ This content is distributed under the terms of the Creative Commons Attribution 4.0 International license.

S. elongatus forms a monophyletic group in the *Synechococcus-Prochlorococcus* clade, with the members of this species clustering separately from other *Synechococcus* species (Fig. 1B; Fig. S2). Within this monophyletic group, there are two groups of strains that have been published as S. elongatus, those strains were isolated from California and Texas, and two *Synechococcus* spp. were recently isolated from Powai Lake in India (28, 29). Although the Indian isolates were named S. elongatus in publication, they share a sequence identity of only ~83% with PCC 7942, well below the 95% threshold for species relatedness (30), and they do not share the identical 16S rRNA gene sequence of the California and Texas strains (Data Set S3). These isolates broaden the phylogenetic branch of this unique group of freshwater *Synechococcus* but were not included in our analysis because of the narrow species-level focus of this study.

10.1128/mbio.00862-22.5 FIG S2 Alignment representation of all UTEX 3055 and legacy strain chromosomes, showing gaps, inversions, and regions of high variability in UTEX 3055 illustrated by SNP density per 1,000 bp. The outer solid ring represents the chromosome alignment of UTEX 3055; the inner solid rings in progression to the center are chromosome alignments of PCC 7942, PCC 7943, PCC 6301, PCC 6311, and UTEX 2973. Download FIG S2, PDF file, 0.9 MB. Copyright © 2022 Adomako et al. 2022 Adomako et al. https://creativecommons.org/licenses/by/4.0/ This content is distributed under the terms of the Creative Commons Attribution 4.0 International license.

### Large-scale genome differences suggest a pattern of laboratory domestication.

**FIG 2:**
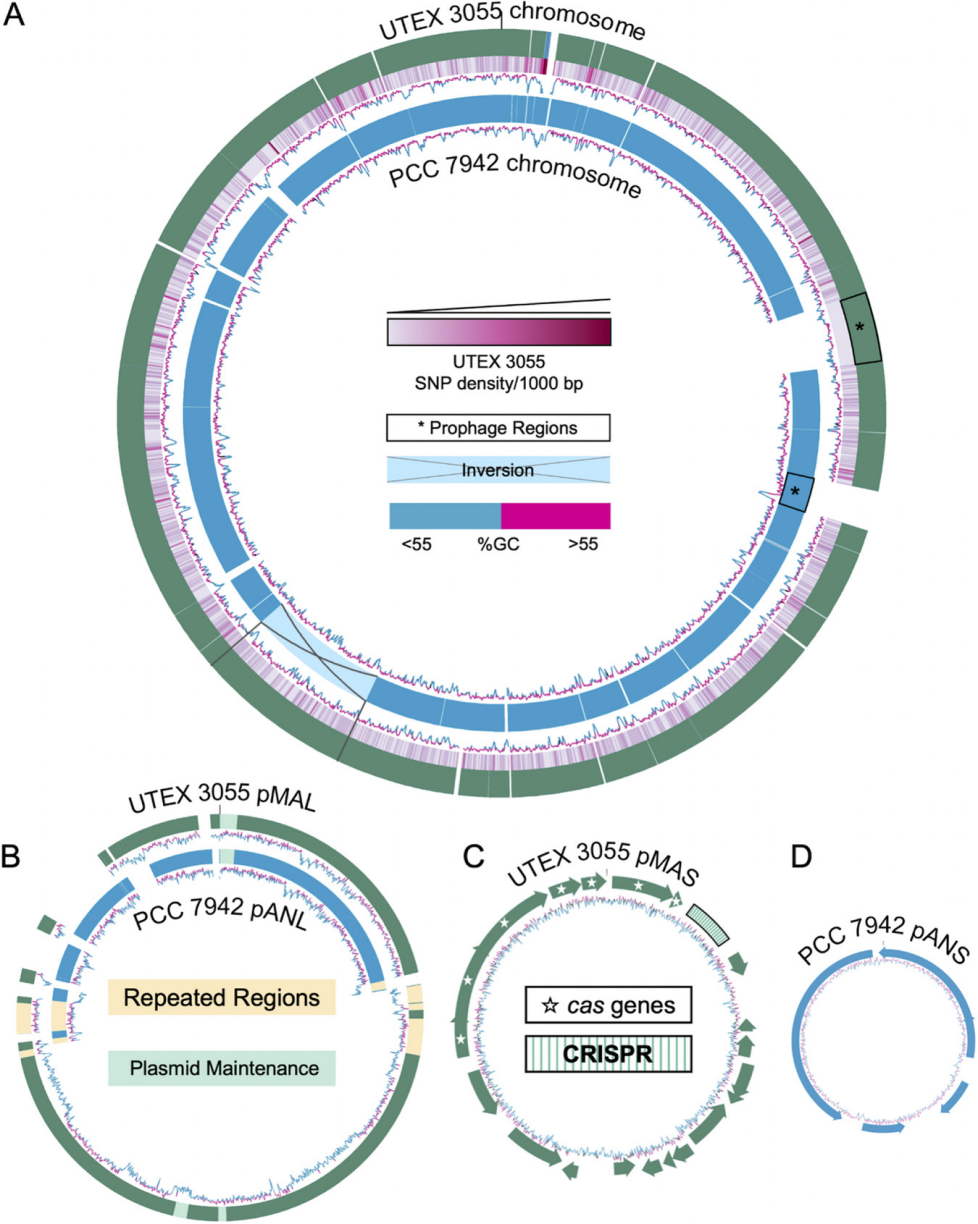
(A) Alignment of PCC 7942 (representing legacy strains) and UTEX 3055 chromosomes showing gaps, inversions, and regions of high variability illustrated by SNP density per 1,000 bp. (B) Alignment of UTEX 3055 pMAL and pANL of legacy strains; repeated regions of homology and plasmid maintenance genes are highlighted. (C and D) Gene maps of the small plasmids UTEX 3055 pMAS (C) and legacy strain pNAS (D).

There are three types of large-scale genomic differences among S. elongatus strains, a chromosomal inversion region, plasmids, and prophage regions (Fig. 2A). A known 188.6-kb inversion is present in PCC 7942 relative to the other strains (19, 26). The sequence of PCC 7943 also contains this inversion (Fig. S2), occurring in the early N-terminal coding region of two porin genes, *somB* and *somB2*, before the predicted conserved porin-domain coding region. Both *somB* and *somB2* contain highly iterated palindrome (HIP1) sequences ahead of the inversion. HIP1 sequences are hyperabundant in cyanobacterial genomes and are implicated in site-specific recombination (31). The inversion does not have any known phenotypic effect but does correlate with a close relationship between PCC 7942 and PCC 7943 that is consistent with the known history of the strains.

The legacy strains of S. elongatus carry a 46.3-kb plasmid (large, pANL) and a 7.8-kb plasmid (small, pANS) that can be cured from the strains (32, 33) (Fig. 2B and D). There is a long history of constructing cyanobacterial shuttle vectors from the backbone of pANS (34), including a self-replicating shuttle vector (35). The large legacy strain plasmid, pANL, has four regions characterized by functions in replication, signal transduction, plasmid maintenance, and sulfur metabolism (36). UTEX 3055 lacks both pANS and pANL, but has two plasmids not seen in the legacy strains, here named pMAS and pMAL. The large plasmid of UTEX 3055, pMAL, is 89.2 kb and shares ~35 kb of homologous content with pANL of the legacy strains (Fig. 2B). The homologous regions of pANL and pMAL include a plasmid maintenance region and the sulfur metabolism cluster of pANL. The experimentally determined replication origin of pANL contains 149-bp direct repeats and overlapping pairs of paralogous open reading frames (ORFs) hypothesized to be the result of duplication or transposition events (36). A region homologous to the replication origin of pANL is found in UTEX 3055 pMAL, followed by a 49.5-kb region with 49 ORFs not homologous to pANL in gene clusters related to sulfonate and heavy metal metabolism, as well as a putative plasmid maintenance region. This expanded region of pMAL is flanked on either side by a duplicated pair of genes (UTEX3055_pgB029/B030; UTEX3055_pgB080/B081) homologous to a pair of genes in pANL (Synpcc7942_B2632/B2633) (Fig. 2B). The homology and synteny with pANL, duplicated genes flanking the expanded region, and the presence of plasmid maintenance genes within the expanded region point to a possible fusion of pANL and another plasmid as the origin of pMAL in UTEX 3055. Site-specific recombination between plasmids at HIP1 sequences has been documented in *Synechococcus* (31), supporting this hypothesis.

The small 24.4-kb plasmid of UTEX 3055, pMAS, contains a plasmid maintenance region, a putative signal transduction region, and a type I-C CRISPR-Cas system (37–39) (Fig. 2C) similar to that of *Synechococcus* sp. PCC 7002, including a similar direct repeat sequence in the CRISPR array (Data Set S3). The spacer sequences were used to search the NCBI nucleotide database and the UTEX 3055 genome for self-targeting spacers, with no significant matches. This outcome is not unexpected, as only a tiny fraction of spacers found in genomic CRISPR arrays can be matched confidently to a protospacer sequence (40). In the pMAS CRISPR-Cas system, Cas4 is fused with Cas1, a common arrangement in several type I systems, but also contains Cas6, which is typically absent from type I-C systems (38). The system may be under the transcriptional control of a WYL domain-containing protein gene directly upstream of the first gene of the system, as a similar transcriptional regulator in *Synechocystis* sp. PCC 6803 negatively regulates a CRISPR-Cas system in that strain (41).

### The legacy prophage controls pigmentation in PCC 7942.

The two largest regions of difference between the legacy strains and UTEX 3055 are prophage regions (Fig. 1A). The legacy strains have a 49-kb insertion not present in UTEX 3055 that was previously described in PCC 7942 and PCC 6301 as encoding a 25-kb cryptic prophage with similarity to marine cyanosiphoviruses (42, 43). Further investigation of this insertion from 711,254 to 759,991 bp (Synpcc7942_0716 to Synpcc7942_0767) in PCC 7942 confirms a prediction made by Phage_Finder (44) that this insertion encodes a 49-kb prophage, which inserted into a tRNA-Leu gene (Synpcc7942_R0040/UTEX3055_pg0872) so that the prophage is flanked by phage attachment (*attL*/*attR*) sites composed of an exact duplication of the last 60 bp of the tRNA-Leu gene. This prophage is completely missing from UTEX 3055, which has a different 89-kb prophage inserted in tRNA-Gly (Synpcc7942_R0032/UTEX3055_pg0587) (Fig. 1A). Similar to the prophage found in legacy strains, the UTEX 3055 prophage was identified through predicted phage genes and flanking duplicate *att* sites. Completely dissimilar to that of the legacy strains, the UTEX 3055 prophage is most similar to the freshwater cyanophage S-EIV1 (45).

**FIG 3:**
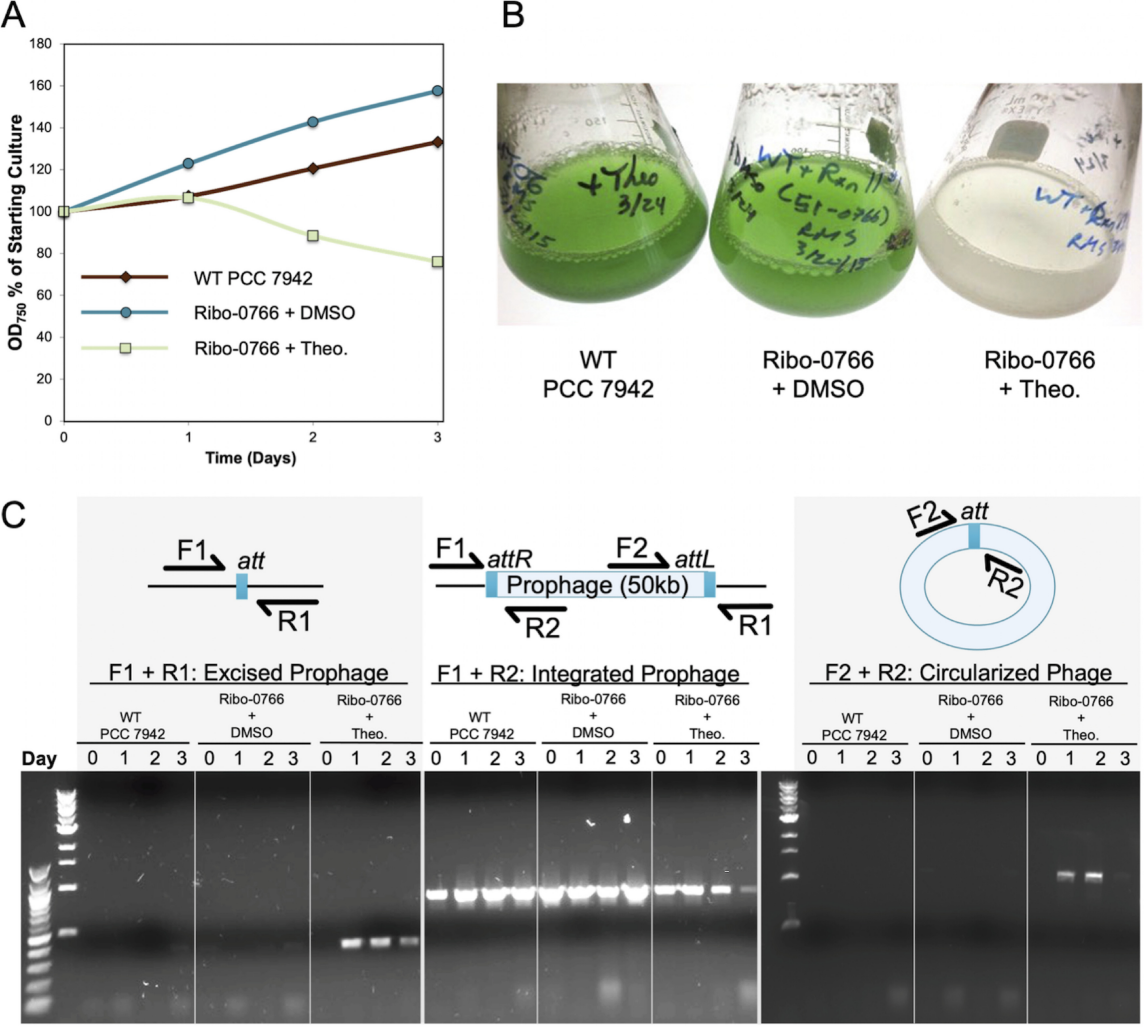
Activation of a theophylline riboswitch driving overexpression of Synpcc7942_0766 induces a loss of culture density (A) and visible lysis (B) by day 3 postinduction. (C) Excision and circularization of the prophage genome following theophylline induction was observed through whole-cell PCR using primer sets with annealing sites just within and outside the prophage region. Cell lysis in induced cultures resulted in faint PCR bands from day 3 samples.

We recognized, in the literature, a strain of PCC 7942 described by Watanabe et al. to be lacking an ~50-kb region covering the majority of the prophage region as a potential prophage excision strain (46). After obtaining this strain from the Yoshikawa lab, named Δ50kb in this work, we verified through PCR and Sanger sequencing that this strain lacks the prophage and possesses only one copy of the *att* site, as would be expected if the prophage excised or had never integrated into this strain. Given the presence of the complete prophage in the other legacy strains, we hypothesized that the prophage may not be cryptic and could excise from the genome. The prophage in the legacy strains encodes a putative Cro/C1-type lytic-lysogenic switch between two divergent operons that each encode putative DNA-binding proteins (Fig. S3). According to published transcriptomic and proteomic data, the lysogenic control operon beginning with Synpcc7942_0764 is actively transcribed and translated under standard laboratory conditions, while the lytic activation operon that includes Synpcc7942_0766, encoding a putative DNA damage-inducible antirepressor, is not (16, 47). Because efforts to induce phage excision through DNA-damaging treatments, including UV irradiation, mitomycin C, and metal toxicity, were not successful, we tested the ability of the prophage to excise through overexpression of Synpcc7942_0766 regulated by a theophylline-inducible riboswitch. Ectopic induction of Synpcc7942_0766 overexpression resulted in a decrease in optical density at 750 nm (OD_750_) after 3 days, while theophylline-treated WT PCC 7942 cultures and uninduced cultures continued to grow (Fig. 3A and B). Microscopic inspection of culture contents and inability of the cultures to regrow indicated that cells had lysed after Synpcc7942_0766 overexpression. A PCR amplification strategy to detect excision and circularization of the phage genome showed circularized phage genomes and prophage-excised chromosomes following induction of Synpcc7942_0766 (Fig. 3C). Although examination of cleared cultures did not reveal phage particles, nor did cell-free fractions enable subsequent rounds of infection in WT PCC 7942, the capacity of the prophage to excise suggests that it is not completely cryptic, though it may require environmental conditions not yet tested in the laboratory (48) or a helper phage for mobilization (49). Prophages often undergo “domestication” by the host genome, losing structural or lytic components while retaining those that are beneficial to the host (50, 51). An example of this type of domestication in the laboratory is exemplified by the deletion of the second *att* site in the prophage region of UTEX 2973 (Fig. S2) that would preclude excision of the prophage from this strain.

10.1128/mbio.00862-22.6 FIG S3 PCC 7942 contains a putative Cro/C1-type lytic/lysogenic switch operon at the right side of the prophage region. The lytic repressor region is actively transcribed in WT PCC 7942, as seen in RNA-Seq data (top graph). Download FIG S3, PDF file, 0.6 MB. Copyright © 2022 Adomako et al. 2022 Adomako et al. https://creativecommons.org/licenses/by/4.0/ This content is distributed under the terms of the Creative Commons Attribution 4.0 International license.

**FIG 4:**
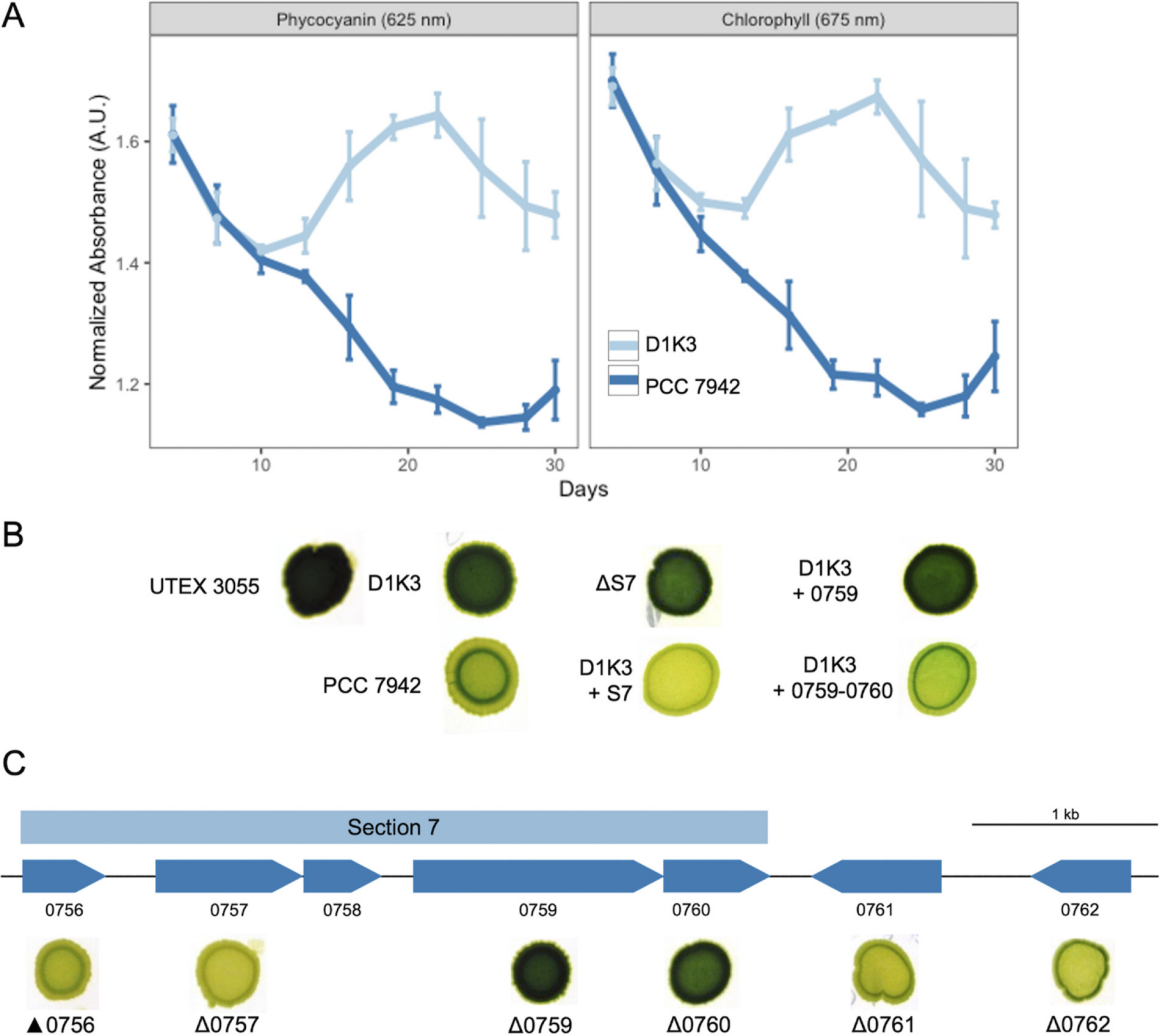
Prophage genes control pigmentation in PCC 7942. (A) Absorbance readings of spot cultures show that PCC 7942 loses phycocyanin and chlorophyll pigments over time compared to the phageless strain D1K3. (B) Loss of either Synpcc7942_0759 or Synpcc7942_0760 in section 7 of the prophage leads to a dark pigmentation phenotype similar to the phageless strain. (C) Expression of both Synpcc7942_0759 and Synpcc7942 _0760 together, but not Synpcc7942_0759 alone in the phageless strain, led to WT light pigmentation.

Although the Yoshikawa lab reported no impact of the lack of the prophage on the growth of Δ50kb compared to WT PCC 7942, we observed that Δ50kb displays a darker appearance upon long-term growth under high light on solid media. Because resequencing of the Δ50kb strain demonstrated that it possesses five SNPs in addition to the phage deletion, we created a clean deletion of the prophage region in our laboratory’s WT PCC 7942. This strain, designated D1K3, has the same dark pigmentation phenotype as Δ50kb (Fig. 4A and B), which time course data indicate is due to the lack of chlorosis that is otherwise observed as decreasing concentrations of phycocyanin and chlorophyll in WT PCC 7942 (Fig. 4A).

UTEX 3055, which does not contain the prophage region of the legacy strains, also has a dark pigmentation phenotype like Δ50kb and D1K3 (Fig. 4B). We hypothesized that the legacy strain prophage encodes genes that regulate the concentration of the photosystem pigments of S. elongatus.

Regions of the prophage with functional similarity and transcriptional orientation were identified and deleted region by region and tested for pigmentation phenotype. Deletion of section 7 (S7), which contains genes encoding a lysozyme and DNA-binding proteins, resulted in the dark pigmentation phenotype of the phageless strains (Fig. 4B). Integration of an S7 amplicon into neutral site I of the S. elongatus chromosome (NS1) resulted in the recovery of the chlorosis phenotype in both the D1K3 phageless strain and the S7 deletion strain, indicating that one or more of the genes present in this region of the prophage are necessary for this phenotype. Genes within S7 were then individually deleted and analyzed, revealing that only deletion of either of the cotranscribed Synpcc7942_0759 or Synpcc7942_0760 genes resulted in a dark pigmentation strain (Fig. 4C). Synpcc7942_0759 and Synpcc7942_0760, respectively, encode a hypothetical protein and a putative restriction endonuclease with high transcription levels under normal laboratory conditions (16). As observed with the S7 complementation, neutral site integration of the Synpcc7942_0759-Synpcc7942_0760 operon under the control of its native promoter recovered the WT light-pigmentation phenotype, though an analogous addition of only Synpcc7942_0759 failed to complement the dark phenotype (Fig. 4B). Attempts to generate a vector that expresses only Synpcc7942_0760 were consistently unsuccessful and may be due to expression of the putative restriction endonuclease selecting against clones in Escherichia coli. Nonetheless, these data demonstrate that either Synpcc7942_0760 alone or in combination with Synpcc7942_0759 regulates the concentration of the photosystem pigments, likely through the degradation of the light-harvesting phycobilisomes and the photosystem complexes. Degradation of phycobilisomes and the subsequent bleaching of cells are mediated in PCC 7942 in response to nutrient limitation (52) by nonbleaching protein A (NblA). Some marine and freshwater cyanophages carry *nblA* genes, presumably favoring the metabolic needs of the phage during a lytic infection (53–55). Synpcc7942_0759 and Synpcc7942_0760 may represent a similar phage strategy of dismantling light-harvesting complexes through a pathway independent of a phage-encoded *nblA*.

### Unique genes in UTEX 3055 are necessary for phototaxis and support a domestication hypothesis for legacy strains.

A gene set enrichment analysis (GSEA) of the set of genes in UTEX 3055 that lack homologs in the legacy strains indicates the genome of UTEX 3055 is enriched in mobilome (mobile genetic elements), defense mechanism, motility, and cell cycle COG-category genes (Fig. S4; Data Set S3). Many genes in the defense mechanism COG category are toxin-antitoxin systems (TAS), which are associated with phage inhibition (56), as well as exposure to diverse environmental stresses (57), where they may be beneficial as stress-response elements for bacteria living in various environments (58). UTEX 3055, as a new environmental isolate, has a more recent history of environmental stress than legacy strains that have been cultivated in controlled laboratory environments for decades. UTEX 3055 has 9 novel TAS not found in the legacy strains and shares 8 of the 11 TAS found in legacy strains. In four of the shared TAS, UTEX 3055 has a deletion or frameshift mutation in the toxin gene of the TAS (Data Set S3), suggesting that these TAS are in the process of being lost. Prokaryotic genomes are shaped by the flux of gene addition via horizontal gene transfer and gene loss, which is a more common mechanism (59). The stasis of TAS in legacy strains compared to the addition and loss of TAS in UTEX 3055 is an indication of how the forces of laboratory domestication do not always lead to loss, as in the case of the prophage *attR* site in UTEX 2973, but can instead stabilize some types of genome elements.

10.1128/mbio.00862-22.7 FIG S4 GSEA of the unique gene set of UTEX 3055 shows an enrichment in mobilome, defense mechanism, motility, and cell cycle COG category genes. Categories that are significantly enriched or depleted are marked with darker category colors and asterisk. Download FIG S4, PDF file, 0.7 MB. Copyright © 2022 Adomako et al. 2022 Adomako et al. https://creativecommons.org/licenses/by/4.0/ This content is distributed under the terms of the Creative Commons Attribution 4.0 International license.

The enrichment of motility genes in the unique gene set of UTEX 3055 is not unexpected, considering its phototactic phenotype. The enrichment in cell cycle genes in UTEX 3055 largely reflects the plasmid maintenance genes of the two plasmids, but further investigation of hypothetical genes listed in this category found two genes, UTEX3055_pg2477 and UTEX3055_pg2478, that are homologous to an operon of *Synechocystis* sp. PCC 6803 necessary for optimal motility and photosystem function (60). In PCC 6803, their gene products may interact with pilus assembly proteins like the type II transport protein GspH. UTEX 3055 has a homolog of GspH (UTEX3055_pg2265) encoded within a four-gene operon (UTEX3055_pg2263-pg2266) that is included in the motility category of the enriched unique gene set. This region nestles within a putative operon for synthesizing nucleotide sugars (61) and contains a homolog of *gspH* and hypothetical genes in motility and extracellular structure COG categories. A protein homology search with Phyre2 (62) predicts that each of the four genes in this cluster encodes similarly to pilin or adhesin domains.

**FIG 5:**
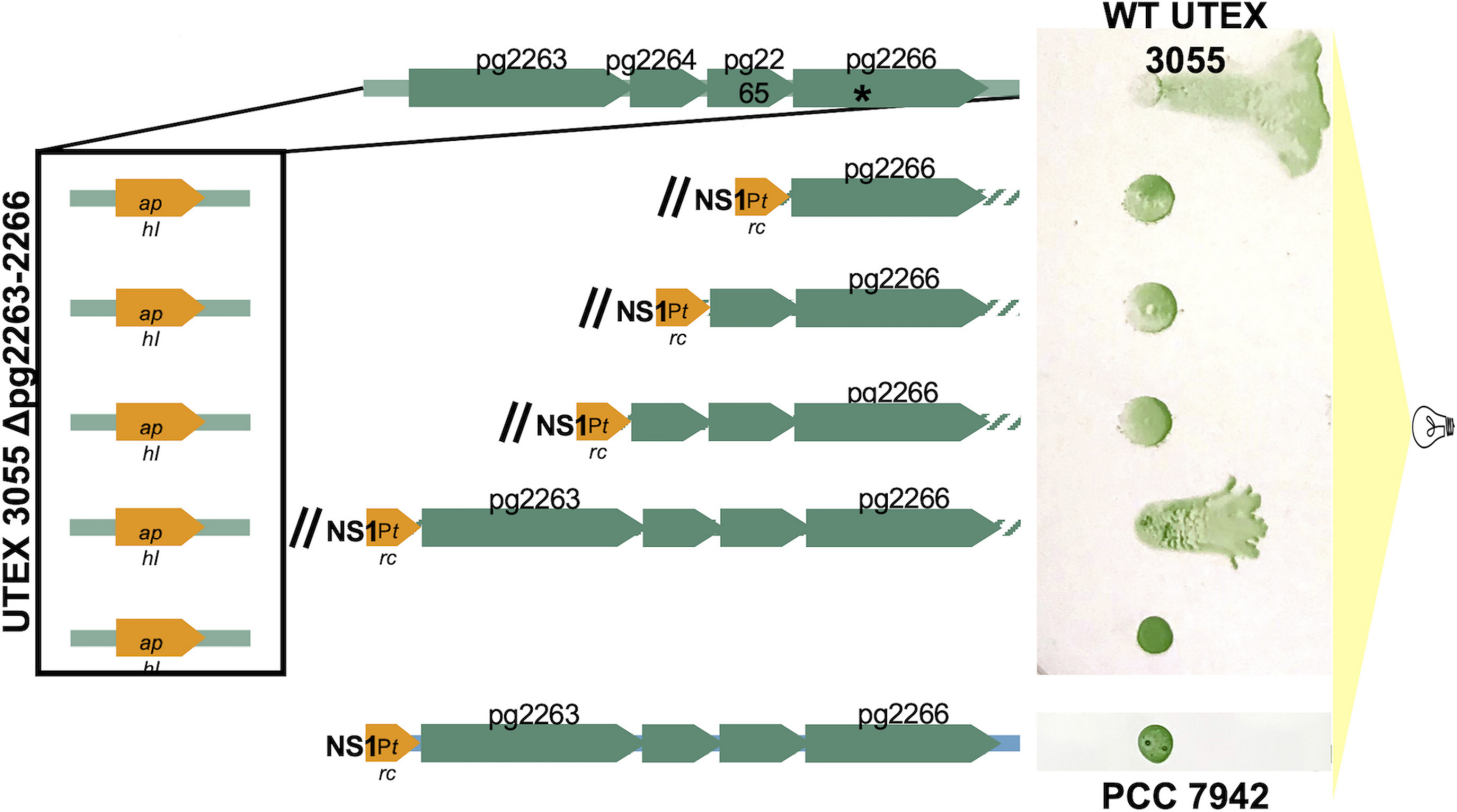
Replacement of UTEX3055_pg2263-pg2266 with a kanamycin resistance cassette (*aphI*) leads to a loss of phototaxis. The site of Tn*5* insertion in the initial phototaxis mutant is marked by an asterisk. Complementation with the full four-gene operon restores phototaxis. Introduction of UTEX3055_pg2263-2266 is not sufficient to restore a phototaxis phenotype to PCC 7942.

In the course of screening a transposon insertion mutant library of UTEX 3055 for phototaxis mutants, we isolated a nonphototactic mutant with an insertion in UTEX3055_pg2266. Because all four genes in the region were hypothesized to have motility functions, the entire region was investigated. We first deleted and replaced the region with a kanamycin resistance cassette through homologous recombination with a mutagenic shuttle vector, and, as expected, this deletion mutant is no longer phototactic (Fig. 5). Four complementation vectors for introduction into a genome-neutral site (Fig. 5) were created to test which of the genes is necessary to restore phototaxis to the deletion mutant. Only addition of the complete novel region restored phototaxis to the deletion mutant (Fig. 5). This suggests that all four genes in the region are necessary for phototaxis in UTEX 3055. The addition of this novel four-gene region in the same neutral genome site of PCC 7942 did not confer phototaxis in PCC 7942 (Fig. 5). In addition to previous findings that PCC 7942 contains a functional photoreceptor and phototaxis operon that is necessary for phototaxis in UTEX 3055 (11), there are likely additional genes necessary for phototaxis in S. elongatus yet to be discovered, and a combination of genes and operons is responsible for the phototaxis phenotype of UTEX 3055.

### SNPs in the pangenome of S. elongatus contextualize strain histories and phenotypes.

The pangenome analysis revealed more than 40,000 SNPs and ~350 indels among the homologous regions of all S. elongatus strains (Supplemental File S2), but only 20% of those SNPs result in amino acid sequence changes in proteins (SNPs relative to PCC 7942 leading to such changes are shown in Table 1). A GSEA analysis of the homologous regions of the pangenome with high sequence conservation between UTEX 3055 and the legacy strains was used to assess what gene categories are fundamental to the fitness of S. elongatus in either environmental or laboratory growth conditions. Homologs with 100% nucleotide sequence conservation are enriched in circadian machinery genes, while homologs with 95% amino acid conservation are enriched in type IV pili machinery, transcription machinery, and energy production genes. These enriched categories are in addition to an enrichment of genes that are conserved across all cyanobacteria and genes that are known essential genes in PCC 7942, underscoring the importance of the circadian and natural competence to S. elongatus, two traits that have made the strain such an attractive model organism.

**TABLE 1:**
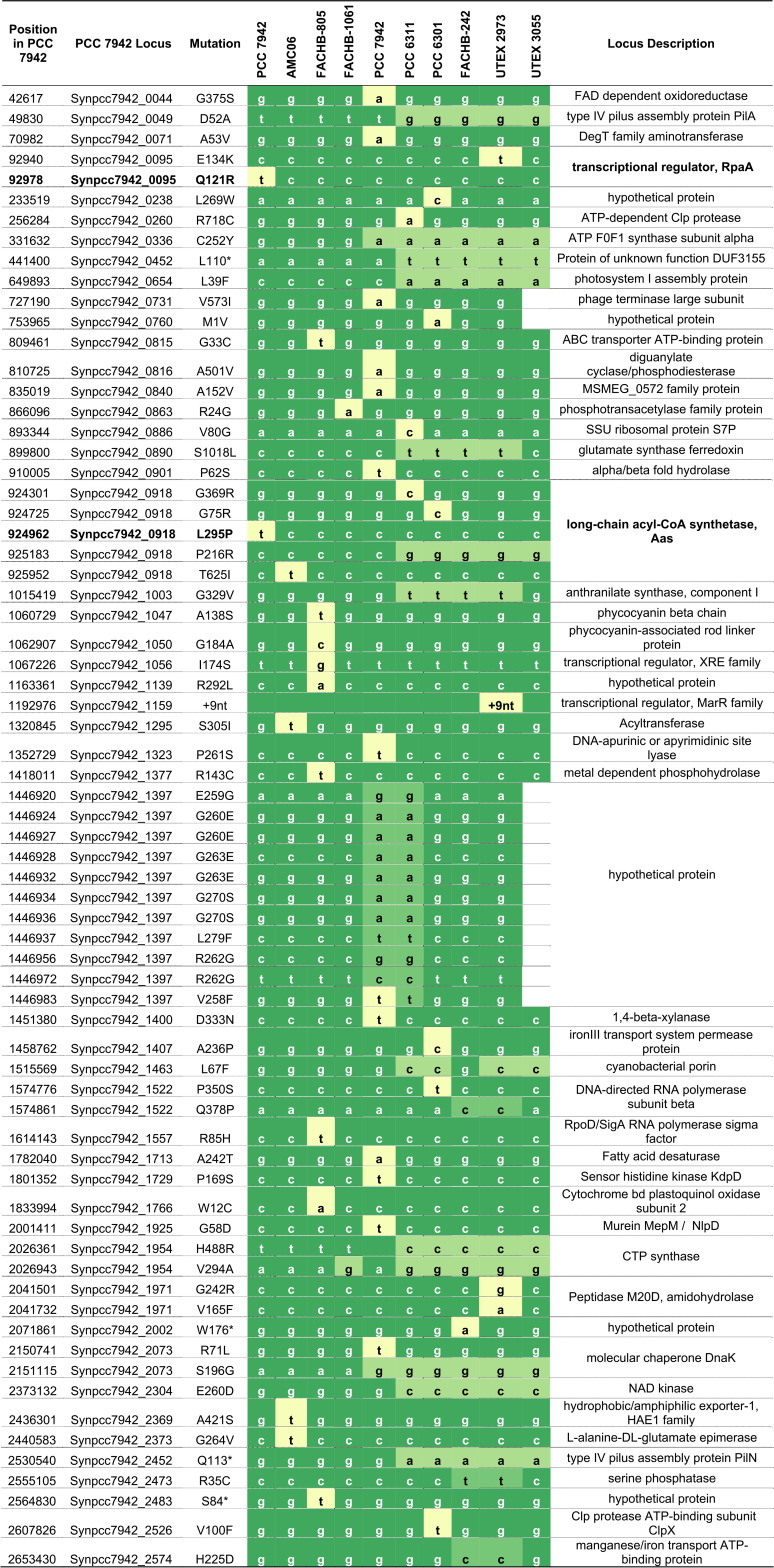
SNPs relative to PCC 7942 that lead to amino acid changes^*a*^

a See Data Set S2 for all SNPs and indels. SNPs shared among several genomes are in light green; SNPs in only one strain are yellow. Two SNPs of the published PCC 7942 sequence (in bold) result in a mutant allele (*rpaA*) and a likely suppressor mutation (*aas*).

10.1128/mbio.00862-22.2 DATA SET S2 All SNPs and indels for the pangenome comparison of S. elongatus strains used in this study. Download Data Set S2, XLSX file, 2.7 MB. Copyright © 2022 Adomako et al. 2022 Adomako et al. https://creativecommons.org/licenses/by/4.0/ This content is distributed under the terms of the Creative Commons Attribution 4.0 International license.

**FIG 6:**
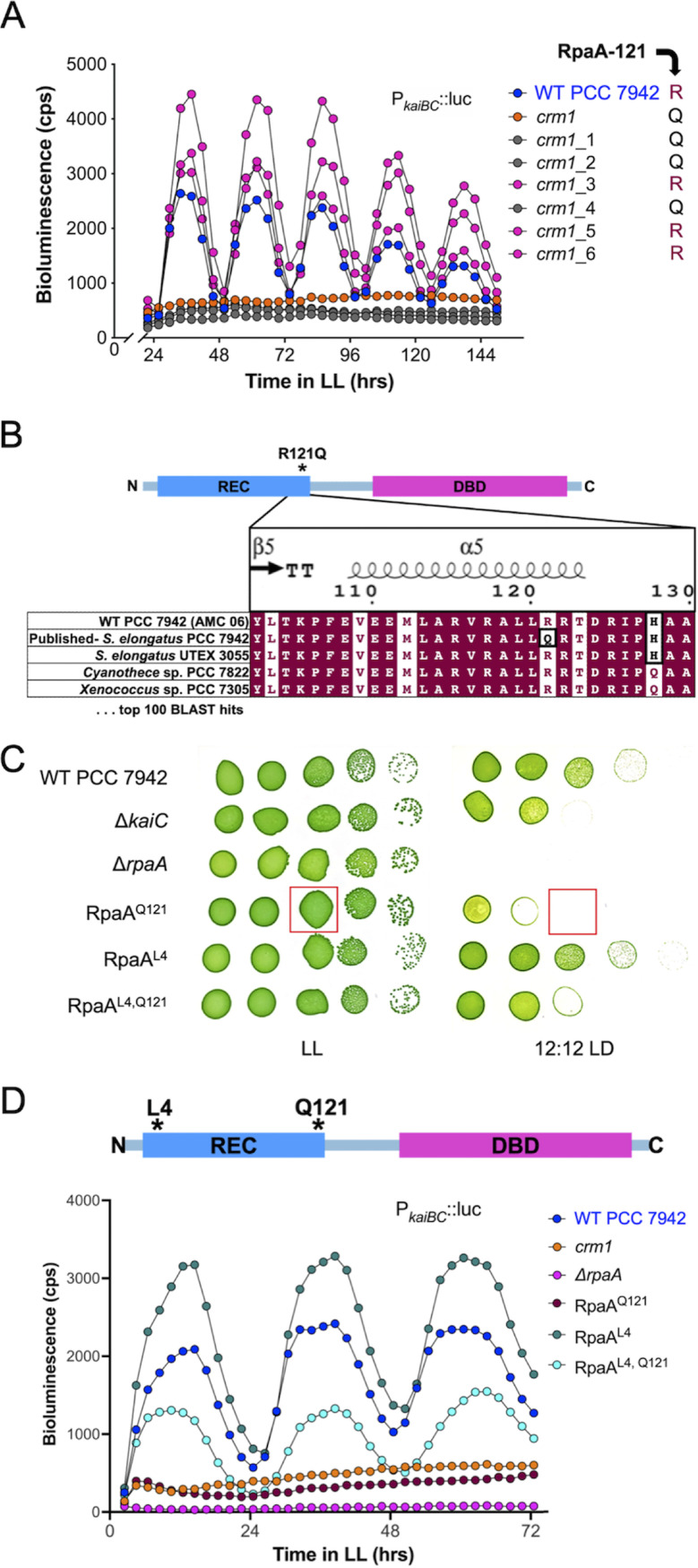
(A) Reconstruction of the *crm1* mutant leads to arrhythmic and rhythmic populations due to the presence (clones represented in gray) or absence (clones represented in magenta) of the RpaA-Q121 allele, respectively. (B) The conserved amino acid at position 121 of RpaA in cyanobacteria is arginine. An RpaA-L4-Q121 suppressor mutation restores LD fitness (C) and rhythmic gene expression (D). Dilution series of strains were grown in constant light (LL) or in 12-h light/dark cycles (12:12 LD) for 48 h to assess LD fitness. Bioluminescence from strains carrying a P*_kaiBC_-luc* reporter at NS2 was recorded as an assay for circadian rhythms of gene expression. LL, constant light after entrainment.

The standardized laboratory culturing conditions that facilitate reproducibility in experiments also present a suite of selective pressures, perhaps unintended, that may shape the genome of S. elongatus, and we hypothesized that examination of the differences among legacy strains could provide insight into these selective pressures. We compared the sequence of a currently propagated culture of PCC 7942 in our lab; a revived culture cryogenically archived in our lab in 1988; our resequence of PCC 6301; recent resequencing data available for PCC 7942 and PCC 6301 archived at the Freshwater Algae Culture Collection at the Institute of Hydrobiology, Wuhan, China (63); the sequences of PCC 6311 and PCC 7943 that are presented in this work; and the previously published genomes of PCC 7942 (GenBank accession no. NC_007604) and UTEX 2973 (GenBank accession no. NZ_CP006471) (Data Set S3). In contrast to the tens of thousands of SNPs present between UTEX 3055 and these strains, there are only 120 SNPs and other differences among all available legacy strain genome data (Data Set S3). The pattern of shared SNPs across legacy strains correlates with the known isolation and archival history of the strains, with the “Texas” strains isolated from Waller Creek (PCC 6301, UTEX 2973, and UTEX 3055) sharing many of the same SNPs (Fig. 6), and clarifies a confusing conclusion about the relationship of UTEX 2973 to PCC 6301 and PCC 7942 (15). *Synechococcus* strain UTEX 2973 was isolated from an archived sample of UTEX 625 (alias, PCC 6301) and was introduced in the literature with a genomic comparison to PCC 6301 and PCC 7942. Yu et al. found ~1,600 SNPs and indels between UTEX 2973 and PCC 6301 but only 55 nucleotide differences with PCC 7942 and concluded that UTEX 2973 is more closely related to PCC 7942 than to PCC 6301, acknowledging that the finding was unexpected considering the history of the strains. Our resequencing of PCC 6301, as well as previously published resequencing of the small plasmid of PCC 7942 pANS (alias, pUH24) (35), shows that the majority of these reported SNPs were unfortunate sequencing errors. The comparative genome analysis with updated sequence information indicates a greater similarity between UTEX 2973 and PCC 6301 that agrees with the strains’ common isolation and cultivation history.

The UTEX 2973 alleles of three genes, ATP synthase subunit alpha *atpA*, NAD^+^ kinase *ppnK*, and the master regulator output of the circadian clock *rpaA*, have been reported to contribute to the fast-growth (or highly light-tolerant) phenotype of UTEX 2973 (20). Of these alleles, *atpA* and *ppnK* are the common alleles among S. elongatus strains, with PCC 7942 as the sole outlier, and Ungerer et al. hypothesize that PCC 7942 has adapted to a low-light lifestyle with these mutations. When the UTEX 2973 sequence of *rpaA* is compared to the published sequence of PCC 7942, there are three differences, an 8-bp deletion in the region upstream of the gene and R121Q and K134E substitutions in the encoded protein. Resequencing of our lab strain PCC 7942 consistently finds four SNPs relative to the published PCC 7942 genome (Data Set S3), one of which is the same R121Q substitution in RpaA reported in UTEX 2973. However, we have found that the WT allele encodes RpaA-R121 in all cyanobacterial strains, and the RpaA-Q121-encoding allele in the published genome of PCC 7942 is present only in the clone used for sequencing. The expectation that the RpaA-Q121-encoding allele is the WT confounded the interpretation of Ungerer et al. on the contribution of the UTEX 2973 allele of RpaA to the fast-growth phenotype and has also caused confusion in previous work in our lab on the genetic network of the circadian clock of S. elongatus.

### One SNP in the circadian response regulator *rpaA* results in an arrhythmic phenotype.

Cyanobacteria are currently the only prokaryotic system with a molecularly described circadian clock, and PCC 7942 is the premier model organism for its study. In cyanobacteria, rhythmic phosphorylation and dephosphorylation of KaiC, a component of the circadian core oscillator, regulates global patterns of gene expression through phosphorylation of the clock output response regulator RpaA. Previously in our lab, a mutant strain of PCC 7942 that lacks rhythmic clock-controlled gene expression was isolated from a transposon mutagenesis screen (64), and the Tn*5* insertion was mapped to a putative open reading frame 358 bp upstream of *rpaA* named *crm* (65). This insertion in *crm* did not phenocopy an *rpaA*-null mutant and had no impact on *rpaA* transcript or protein accumulation, but KaiC abundance and rhythmic phosphorylation were diminished. These results suggested that the *crm1* mutation had no *cis*-regulatory impact on *rpaA* and, instead, perturbed clock-controlled gene expression through an unknown mechanism (65). However, we recently discovered that the phenotypes ascribed to *crm* derive from an unusual allele of *rpaA* (66).

In an effort to understand the role of *crm* in clock-controlled gene expression, the *crm1* insertion allele was reconstructed in a WT background using the mutagenesis cosmid from the original transposon-mutagenesis screen. Of six randomly selected *crm1* clones, three showed WT rhythms of circadian gene expression, and three showed the expected arrhythmic phenotype (Fig. 6A). Sequencing of *rpaA* from these clones showed that the arrhythmic subpopulation contained the RpaA-Q121 allele matching the published genome of PCC 7942. The rhythmic subpopulation contained an apparent mutant allele of *rpaA* with arginine at position 121 (RpaA-R121); however, BLAST results of 100 cyanobacterial RpaA homologs show universal conservation of arginine at this position (Fig. 6B), and WT PCC 7942 strains resequenced in our lab also encode the conserved arginine residue. The conservation of arginine at this position among cyanobacterial homologs of RpaA, the appearance of the conserved arginine residue in rhythmic WT PCC 7942 strains, and the arrhythmic phenotype of RpaA-Q121 show that RpaA-R121 is the true WT allele of PCC 7942.

From this evidence, we discovered that the single colony of PCC 7942 from our lab that was sequenced by JGI and published in GenBank, and also used to construct a UniGene Set (UGS) mutant library (64), carried the arrhythmic RpaA-Q121 allele. The UGS cosmid containing the *crm1* transposon insertion also carries the complete coding sequence of the mutant allele *rpaA*-Q121, and so, mutants constructed using this cosmid can contain either the arrhythmic RpaA-Q121 or the WT (rhythmic) RpaA-R121, depending on where crossovers occur during homologous recombination (Fig. S5). This variable resulted in the two subpopulations observed when reconstructing *crm1* mutants for this study and explains the arrhythmic phenotype of the original *crm1* mutant as a result of the incorporation of the arrhythmic *rpaA*-Q121 allele, rather than a transposon insertion in *crm1*. The *rpaA*-Q121 allele was not recognized in the original *crm1* study because of apparent complementation of the arrhythmic *crm1* (i.e., RpaA-Q121) phenotype to rhythmicity, which was attributed to ectopic expression of full-length *crm* (65). Follow-up experiments indicated that the RpaA-Q121 (*crm1*) mutant was poorly transformable. Because the expression of certain essential competence genes in PCC 7942 is under the control of phosphorylated RpaA (26), we infer that selection for a transformant during complementation studies would likely also select for reversion that restored RpaA function. Indeed, full-genome sequencing of the *crm*/*crm1*-complemented strain revealed a second-site suppressor mutation in *rpaA*, RpaA-L4-Q121.

10.1128/mbio.00862-22.8 FIG S5 Homologous recombination events of the WT PCC 7942 chromosome with the UGS *crm*:Tn*5* disruption cosmid can produce kanamycin-resistant clones that are either rhythmic and carry WT *rpaA* or are arrhythmic by assimilating *rpaA^G362A^*. Download FIG S5, PDF file, 0.1 MB. Copyright © 2022 Adomako et al. 2022 Adomako et al. https://creativecommons.org/licenses/by/4.0/ This content is distributed under the terms of the Creative Commons Attribution 4.0 International license.

The phenotypic relationship between the RpaA-Q121 arrhythmic and RpaA-L4 suppressor mutations was confirmed by reconstructing *rpaA* mutation combinations via CRISPR-Cas12a engineering (66). In addition to its role as the transcriptional response regulator of the circadian clock, RpaA plays a critical role in redox management, and *rpaA*-null mutants become inviable in darkness (67). The RpaA-Q121 mutant strain is arrhythmic and sensitive to light-dark (LD) cycles, but RpaA-L4 has WT rhythms and LD survival (Fig. 6C and D). The combination of the two substitutions, RpaA-L4-Q121, restored rhythms and improved LD tolerance, confirming the RpaA-L4 mutation as a suppressor of the arrhythmic phenotype of RpaA-Q121. *In vitro* studies of the circadian oscillator show that as RpaA is rhythmically phosphorylated and dephosphorylated in its role as the output of the circadian clock, it rhythmically binds to DNA. The RpaA-Q121 mutant binds DNA poorly despite having a WT phosphorylation pattern; the Q121 substitution prevents the phosphorylated sensor domain of the protein from regulating the DNA-binding domain (66). It is possible that the RpaA-L4-Q121 suppressor mutant restores rhythmic gene expression patterns by restoring the ability of the sensor domain to regulate the DNA-binding domain, subsequently restoring rhythmic gene expression and LD tolerance.

We propose that in the previous *crm1* study, repeated exposure to LD transition events and selection for transformation as part of complementation tests provided a selection for the RpaA-L4-Q121 suppressor mutant. We leveraged the LD sensitivity of RpaA-Q121 in a selection strategy to identify additional suppressor mutations in search of new genes associated with the circadian system. The RpaA-Q121 mutation was introduced into a PCC 7942 containing a reporter of circadian gene expression, and diluted cultures were plated and grown in LD cycles. Colonies that emerged from the LD selection were then screened for rhythmic gene expression. Ten mutants that showed both improved rhythmic gene expression and LD tolerance were chosen for whole-genome sequencing (Fig. S6A and B). Of them, one mutant contained a second-site SNP mutation in the promoter region of *rpaA*, and the eight mutants had second-site mutations in either *clpX* or *labA* (Fig. S7; Data Set S3), genes that have been shown previously to have roles that are not well understood in the mechanism of the circadian clock. *LabA* is required for negative feedback regulation of the core oscillator component KaiC and has been shown to modulate RpaA function (68). RpaA directly regulates *clpX* expression, and the protein degradation activity of the ClpXP protease fine-tunes the circadian clock (69). The 10th mutant from the LD selection was sequenced but did not actually have improved rhythmic gene expression. This arrhythmic but LD-tolerant mutant had a second-site mutation in leucyl peptidase, part of the pathway that recycles glutathione, an important antioxidant that helps maintain the redox balance in cyanobacteria (70). This suppressor screen did not find additional genes in the circadian clock network but reinforced the roles of components that fine-tune the clock mechanism, especially as it relates to maintaining the redox balance of the cell.

10.1128/mbio.00862-22.9 FIG S6 (B) The arrhythmic PCC 7942 RpaA-R121Q CRISPR/Cpf1-edited strain DEC45 was selected for suppressors that restore LD survival. (A) Strains that emerged from this selection were screened for restoration of the rhythmic phenotype. Dilution series of strains were grown in constant light (LL) or in 12-h light/dark cycles (12:12 LD) for 48 h to assess LD fitness. Bioluminescence from strains carrying a P*_kaiBC_-luc* reporter at NS2 was recorded as an assay for circadian rhythms of gene expression. LL, constant light after entrainment in 12:12 LD. Download FIG S6, PDF file, 0.6 MB. Copyright © 2022 Adomako et al. 2022 Adomako et al. https://creativecommons.org/licenses/by/4.0/ This content is distributed under the terms of the Creative Commons Attribution 4.0 International license.

10.1128/mbio.00862-22.10 FIG S7 Nine of 10 suppressor mutants of RpaA-Q211 show restored rhythmic gene expression. Bioluminescence from strains carrying a P*_kaiBC_-luc* reporter at NS2 was recorded as an assay for circadian rhythms of gene expression. LL, constant light after entrainment in 12:12 LD. Download FIG S7, PDF file, 0.8 MB. Copyright © 2022 Adomako et al. 2022 Adomako et al. https://creativecommons.org/licenses/by/4.0/ This content is distributed under the terms of the Creative Commons Attribution 4.0 International license.

The results of this screen and the presence of second-site mutations in transformants of RpaA-Q121 mutants may also help explain some SNPs between PCC 7942 and UTEX 2973. In addition to the RpaA-Q121 allele, the published sequence of PCC 7942 contains an SNP in the long-chain fatty acid CoA ligase gene (*aas*), which plays a critical role in fatty acid recycling (71–73), resulting in the allele *aas*-L295. Like RpaA-Q121, this mutation, Aas-L95, is present only in the published sequence of PCC 7942 and not in any other cyanobacterial homologs of *aas*, including that of an archived sample of our lab strain cryopreserved in 1988, and we propose that Aas-L295 is also a second-site repressor of RpaAQ121. Fatty acid accumulation is seen in *rpaA*-null mutants of PCC 7942 (67), possibly as a result of redox crisis, and the Aas-L295 mutation may mitigate the effects of this accumulation. In UTEX 2973, there are two unique differences in RpaA, a deletion 107 bp upstream of the start codon of *rpaA* and a K134E substitution. However, the effect of these unique differences on the highly light-tolerant and fast-growth phenotype of UTEX 2973 cannot be determined from the current published data due to the use of the RpaA-Q121 allele (20, 74). The unique *rpaA* allele of UTEX 2973 was not compared to a true wild-type *rpaA* allele but, rather, to an arrhythmic, noncompetent, and LD-sensitive mutant. Additionally, our work shows that transformants of *rpaA*-Q121 mutants will likely contain second-site mutations that restore competence. With the context provided by this work, the true contribution of the UTEX 2973 *rpaA* allele can now be determined without the confounding data provided by an unfortunate mutant clone sequenced more than 15 years ago.

### Conclusions.

This work paves the way for improved future genomic analysis in S. elongatus by correcting the PCC 6301 genome sequence and bringing it closer to the sequences of the legacy strains, specifically to UTEX 2793 that is presumably derived from it. It also explains the genetic basis of the *crm1* arrhythmic mutant of PCC 7942, previously attributed to an ORF upstream of *rpaA*, but, in fact, deriving from an allele that is neither WT nor a sequencing error but deriving from a rare mutant clone used for the published reference sequence for PCC 7942.

The comparative genomics analysis identified specific loci that explain a difference in pigmentation and phototaxis phenotypes between UTEX 3055 and the legacy strains. The patterns of shared and unique SNPs and genes between UTEX 3055 and the legacy strains are compatible with a domestication hypothesis; the repeated passage of laboratory cultures by pipetting or pouring would favor planktonic cells that do not form biofilms and may have led to an early selection among the legacy strains, resulting in a planktonic phenotype. In the absence of biofilms, there would be no selection for phototaxis, a phenotype also missing from legacy strains. These patterns of differences will aid the future discovery of additional genes responsible for the phenotypic differences between strains. For example, the model strain PCC 7942 has been actively curated for its facile genetic manipulation in the lab and efficient transformation; in direct comparisons, UTEX 3055 was consistently ~100× less efficient in transformation than PCC 7942. One potential source of reduced transformation in UTEX 3055 is its CRISPR-Cas system that is not present in the legacy strains, and disabling this system could lead to more efficient genetic manipulation of UTEX 3055 in the lab. Another pattern of difference between UTEX 3055 and the legacy strains is an enrichment in unique motility COG category genes, including genes related to exopolysaccharide synthesis. This enrichment is consistent with the biofilming and phototactic phenotype of UTEX 3055, and investigation of this gene set may reveal the genetic basis of biofilm formation and phototaxis in S. elongatus.

## MATERIALS AND METHODS

### Whole-genome alignment and pangenome annotation analysis.

The following genomes of S. elongatus PCC 7942 (GenBank accession nos. NC_007604, NC_007595, and KT751091), PCC 6301 (NC_006576 [previous]; CP085785), PCC 6311 (GenBank accession no. CP088958-60), PCC 7943 (GenBank accession no. CP088961), UTEX 2973 (GenBank accession no. CP006471), and UTEX 3055 (NZ_CP033061) were used for whole-genome alignment. Chromosomes and plasmids were separately aligned using Mauve (75), and the alignment was manually inspected and adjusted using the Mauve plugin in Geneious Prime 2020.1.2 (https://geneious.com). SNPs, gap locations, and ortholog groups were exported from Mauve and further analyzed with a custom R script and are available in Supplemental File S2 in the supplemental material. Core and pangenome elements were determined using full-genome alignment and ortholog analysis. Hypothetical proteins in regions of interest were examined further using PSI-BLAST (76) searches for homologs and Phyre2 to search for homologous protein domain architectures (62). Ortholog assignments from Mauve were further refined using Pfam and COG category analysis in eggNOG-mapper (77, 78). These ortholog assignments were checked against homology groups created through reciprocal nucleotide BLAST search using Vespa (79). Homology groups were translated and realigned using MAFFT (80) in Geneious Prime, and the percentages of identical residues for the nucleotide and amino acid alignments were reported. Annotations were adjusted using annotation consensus agreement, RNA-Seq data (16, 17), and PCC 7942 essentiality data (18) by use of custom R scripts. Gene metadata, including previously used locus tags, pangenome annotations, and gene categorical information such as known biofilm genes from previous S. elongatus PCC 7942 biofilm publications (81–85), pili genes described by Taton et al. (27), essentiality and conservation data from Rubin et al.(18), and functional categories from Pfam and COG category analysis, were amassed for all genes in the S. elongatus pangenome and are provided in Supplemental File S1.

### Average nucleotide identity, average amino acid identity, and phylogenetic tree analysis.

Average nucleotide identity (ANI) and average amino acid identity (AAI) of whole genomes were obtained using online tools (http://enve-omics.ce.gatech.edu/) (86, 87). A phylogenetic tree was built using 29 conserved housekeeping genes previously defined for bacterial multilocus sequence analysis (MLSA) (88). In addition to the 6 S. elongatus strains, 32 additional cyanobacteria were used to build the tree; Prochlorothrix hollandica PCC 9006 was used as the outgroup. Each of the 29-gene sets was aligned using the MAFFT (80) algorithm in Geneious and trimmed using trimAl version 1.2 (89) using the “automated1” option optimized for maximum-likelihood tree construction. The resulting trimmed, aligned files were concatenated and processed using FastTree version 2.1.11 (90) in Geneious. The tree was visualized with FigTree version 1.4.4.

### Bacterial strains and growth conditions.

The strains used in this study are described in Data Set S3. S. elongatus PCC 7942 and UTEX 3055 and their derivative strains were grown in BG-11 medium (91) as liquid cultures with continuous shaking (125 rpm) or on agar plates (40 mL, 1.5% agarose) at 30°C under continuous illumination of 100 to 150 μmol photons m^−2^ s^−1^ from fluorescent cool white bulbs. Culture media for recombinant cyanobacterial strains were supplemented as needed with 2 μg mL^−1^ spectinomycin (Sp) plus 2 μg mL^−1^ streptomycin (Sm), 2 μg mL^−1^ gentamicin (Gm), 7.5 μg mL^−1^ chloramphenicol (Cm), and 5 μg mL^−1^ kanamycin (Km). S. elongatus PCC 6301, PCC 6311, and PCC 7943 were grown at the Pasteur Culture Collection (PCC) at 6 μmol photon m^−2^ s^−1^ at 25°C in liquid BG11 medium. PCC 6301 was revived from a 1988 frozen archive in the Susan Golden lab. PCC 6311 and PCC 7943 were used from alive and axenic cultures at the PCC.

### Full-genome sequencing.

In preparation for full-genome sequencing, S. elongatus PCC 6301, PCC 6311, and PCC 7943 cultures were centrifuged, and the cell pellets were rinsed twice with sterile water and then freeze-dried and lyophilized prior to DNA extraction. For PCC 6301, genomic DNA was extracted with the NucleoBond genomic DNA purification kit (Macherey-Nagel) as previously used for various pure cyanobacteria (92). For PCC 6301 sequencing, a DNA library was prepared using the NextFlex PCR-free DNA sequencing kit (Bioo Scientific, USA) following the manufacturer's recommendations. The library was sequenced on a HiSeq 2000 platform (Illumina, San Diego, CA, USA) in paired-end reads of 101 bases. Sequence files were generated using Illumina Analysis Pipeline version 1.8 (CASAVA; Illumina). After quality filtering with Institut Pasteur in-house bioinformatic tools, 25,191,450 reads were analyzed using CLC Assembly Cell 4.4.0 and CLC Genomics Workbench 7.5.1 (CLC Bio, Qiagen); 20,073,488 paired reads were mapped on the genome sequence of the strain PCC 6301 with CLC Assembly Cell 4.4.0 with an average coverage of 917×. The two plasmid sequences were assembled using Genomics Workbench 7.5.1. For PCC 6311 and PCC 7943, the Illumina sequencing service at GATC (GATC Biotech SARL, Mulhouse, France) was used to generate genome sequences for PCC 6311 (10 scaffolds) and PCC 7943 (9 scaffolds). The genome scaffolds were further assembled into complete chromosome and plasmid sequences using full-genome alignment comparison to PCC 7942 in Mauve. The PCC 6301 sequence is deposited in GenBank under accession numbers CP085785 to CP085787. The PCC 6311 and PCC 7943 sequences are deposited in GenBank under accession numbers CP088958 to CP088960 and CP088961 to CP088963.

### Gene set enrichment analysis.

Categorical metadata available from multiple sources for PCC 7942 were curated for all genes in the pangenome and include essentiality and conservation data (18), pili and competence genes (27), and known biofilm genes (10, 81–84), along with the functional categories (COGs) determined in the pangenome analysis. Gene sets of interest in the pangenome were identified, and significant enrichments in metadata categories for these gene sets were determined using custom R scripts. Briefly, enrichment values were determined using two-sided Fisher’s exact tests, with false-discovery rate (FDR)-adjusted *P* values of ≤0.05 being designated significant. Fold enrichment (*F*) was calculated as the number of genes in the pangenome interest group that are also in the metadata category (*N*_gc_) divided by the number of genes expected in the group and category (*E*_gc_). This expected number was calculated by multiplying the number of total genes in the pangenome interest group (*N_g_*) by the frequency of all genes in the genome that are found in the metadata category (*f_c_*), which was determined as the number of genes in the category (*N_c_*) divided by the number of genes in the genome (*N*).

$$F\;=\;N_{\text{gc}}/E_{\text{gc}};\;E_{\text{gc}}=\;N_{g}\times \;f_{c};\;f_{c}=\;N_{c}/N$$

### Construction of knockout and complementation strains.

Recombinant strains of S. elongatus were constructed by natural transformation using standard protocols (93). Excepting RpaA point mutations, cyanobacterial mutants were generated by transforming with knockout vectors engineered with the CYANO-VECTOR assembly system (94) or transposon insertion vectors from the PCC 7942 UniGene Set (UGS) library (64, 95). To generate D1K3, a multistep approach was used: the prophage was first tagged at neutral site 3 (NS3), located within the prophage, with a counterscreenable antibiotic-resistance cassette (SpSm) and a counterselectable marker, *sacB*, which results in cell death in the presence of sucrose. This tagged strain was then transformed with a prophage deletion vector and selected on plates containing sucrose. Complementation and riboswitch expression strains were constructed by expressing gene(s) ectopically in S. elongatus chromosomal neutral sites NS1 and NS2 (43, 93). Complete segregation of the mutant loci was PCR verified. Unless described otherwise, plasmids were constructed using the GeneArt seamless cloning and assembly kit (Life Technologies) and propagated in Escherichia coli DH5α or DB3.1 with appropriate antibiotics. E. coli strains were grown at 37°C in lysogeny broth (LB; Lennox) liquid culture or on agar plates, supplemented as needed with 100 μg mL^−1^ ampicillin (Ap), 20 μg mL^−1^ Sp plus 20 μg mL^−1^ Sm, 15 μg mL^−1^ Gm, 17 μg mL^−1 ^Cm, and 50 μg mL^−1 ^Km. The plasmids used in this study are described in Data Set S3.

### Phage lysis and pigmentation assays.

Strains were grown in liquid with continuous shaking as previously described to OD_750_ of approximately 0.8 to 0.95. Strains were induced with 2 mM theophylline (200 mM stock dissolved in 100% dimethyl sulfoxide [DMSO]) or 1% DMSO as a control. For 3 days following induction, OD_750_ was measured daily, and colony PCR was used to determine excision of phage genome. Primers used are in Data Set S3. For pigmentation assays, strains were grown in liquid BG-11 for 3 to 4 days to an OD_750_ of approximately 0.5, and 4-μL samples of culture were spotted on BG-11 agar and grown at 30°C under continuous illumination of 300 μmol photons m^−2^ s^−1^ for 30 days. Strains were spotted on the plates in grids to facilitate visual comparison of pairs of strains. For measurement of chlorophyll and phycocyanin content, spots were scraped and resuspended in 200 μL of BG-11. Samples were measured using a Tecan plate reader at OD_625_ for phycocyanin and OD_675_ for chlorophyll and normalized for cell density by dividing by OD_750_. In cases where resuspensions were too dense to accurately measure, resuspensions were diluted 1:1 or 1:2 with BG-11 as necessary and then measured again.

### UTEX 3055 mutant library screening.

A small Tn*5* mutant library in S. elongatus UTEX 3055 was constructed in a similar manner as previously described for PCC 7942 (18). Briefly, UTEX 3055 was grown in liquid culture as previously described until it reached OD_750_ of 0.5. A diaminopimelic acid (DAP) auxotrophic E. coli donor strain carrying a library of barcoded Tn*5* elements (pKMW7) (96) was grown in LB broth with 60 μg/mL DAP and 50 μg/mL kanamycin to an OD_600_ of 1.0. Both E. coli and S. elongatus cells were washed twice and resuspended in BG-11 supplemented with 5% LB at a 1:1 donor cell/recipient cell ratio and spotted on BG-11 with 5% LB agar plates with 60 μg/mL DAP. The conjugation reaction was performed for 12 h under 40 μmol photons·m^−2^·s^−1^ of illumination and then resuspended in BG-11 and plated onto BG-11 Km agar plates for selection of exconjugants. After 10 days of growth under 100- to 140-μmol photons·m^−2^·s^−1^, colonies were patched onto BG-11 Km agar plates. To screen for phototaxis mutants, strains were struck onto BG-11 medium with 10 mM sodium thiosulfate solidified with 0.3% agarose (wt/vol), and the plates were placed in a dark box with one side opening toward a fluorescent light, and phototactic movement was assessed after 3 days. Strains were screened twice, and confirmation of the phototaxis phenotype was performed on 2-μL samples of culture adjusted to OD_750_ of 0.6 to 1.0 spotted at specific positions on the surface of agarose plates and grown as described to assess phototaxis. The insertion location of the transposon in selected mutant strains was determined by colony PCR with arbitrary primers (Data Set S3) and Sanger sequencing.

### Construction of RpaA mutant strains.

The introduction of point mutations into the S. elongatus chromosome was accomplished using a previously described CRISPR-editing approach (97) (66). Briefly, the pSL2680 (Km^r^) plasmid used for CRISPR-Cas12a (formerly Cpf1) editing was purchased from Addgene (Plasmid #85581). Forward and reverse primers upstream and downstream of the desired mutation were annealed together and ligated into AarI-cut pSL2680 to serve as the gRNA template. The resulting construct was purified and digested with KpnI to facilitate insertion of the homology-directed repair (HDR) template. The HDR template was generated by amplifying overlapping upstream and downstream fragments containing the desired point mutations. The upstream and downstream HDR fragments were assembled into KpnI-cut pSL2680 plus genomic RNA (gRNA) using the GeneArt seamless assembly kit (Thermo Fisher Scientific). The plasmids used in this study are described in Data Set S3.

Editing plasmids were electroporated into E. coli DH10B containing helper plasmid pRL623 and conjugal plasmid pRL443 (94). The resulting strain was grown overnight in LB medium containing antibiotics, washed 3× with fresh LB, and mixed in a 1:2 ratio with an S. elongatus reporter-strain aliquot. The cell mixture was plated onto BG-11 agar with added LB (5% [vol/vol]), incubated under 100 μmol m^−2^ s^−1^ light for 36 h and then underlaid with Km (10 μg/mL final concentration) to select for S. elongatus cells that contain the editing plasmid. Colonies that emerged after 6 to 8 days were passaged three times on BG-11 agar containing Km to allow editing to occur. Successful editing of chromosomal rpaA was verified by sequencing. Plasmids were cured from the edited strains by inoculating cells into nonselective BG-11 medium, growing the culture to an OD_750_ of 0.6, and then dilution plating on nonselective BG-11 plates. Fifty colonies were picked and replica patched to selective (Km) and nonselective medium to identify and isolate clones that had lost the editing plasmid.

### Suppressor screens and circadian bioluminescence monitoring.

A flask of DEC45 (*rpaA*-Q121) was grown to OD_750_ of ~0.8, diluted 1:100, and plated (100 μL per plate) across 10 BG-11 agar plates. Plates were grown in LD 12:12 (200 uE) for 7 to 10 days. We inoculated 384/~600 colonies that emerged from the LD selection into 200 μL of BG-11 media in 96-well plates. Bioluminescence was monitored using a P*kaiBC::luc* firefly luciferase fusion reporter inserted into a neutral site of the S. elongatus chromosome as previously described (98). Strains to be monitored were grown in liquid culture to OD_750_ values of of 0.4 to 0.7, diluted to OD_750_ of 0.2, and added as 20-μL aliquots to 280 μL of BG-11 agar containing 3.5 mM firefly luciferin arrayed in 96-well plates. Plates were covered with a gas-permeable seal, and cells were entrained under 12-h light-dark cycles (80 μmol m^−2^ s ^−1^ light) to synchronize clock phases. After 48 h of entrainment, cells were released into continuous light (30 μmol m^−2^ s ^−1^), and bioluminescence was monitored every 2 h using a Tecan Infinite Pro M200 bioluminescence plate reader. Data were collected and plotted using GraphPad Prism 8, with each plot representing the average of six biological replicates. Data were analyzed for rhythmicity using the JTK_CYCLE method provided by BioDare 2 (99, 100) (https://biodare2.ed.ac.uk). LD-tolerant suppressors displaying periodic bioluminescence production were scaled up in flasks for follow-up studies and whole-genome sequencing (WGS).

### Data availability.

Genome annotation and SNP data sets are provided in the supplemental material for the paper. Genome sequences and annotations generated in this study are available in GenBank under accession numbers CP085785 to CP085787, CP088958 to CP088960, and CP088961 to CP088963.
