# Supplementary figures and images for: Comparative Genomics of Synechococcus elongatus Explains the Phenotypic Diversity of the Strains
Source: mBio. 2022 Apr 27;13(3):e00862-22. doi: 10.1128/mbio.00862-22 (PMC9239245; doi:10.1128/mbio.00862-22)

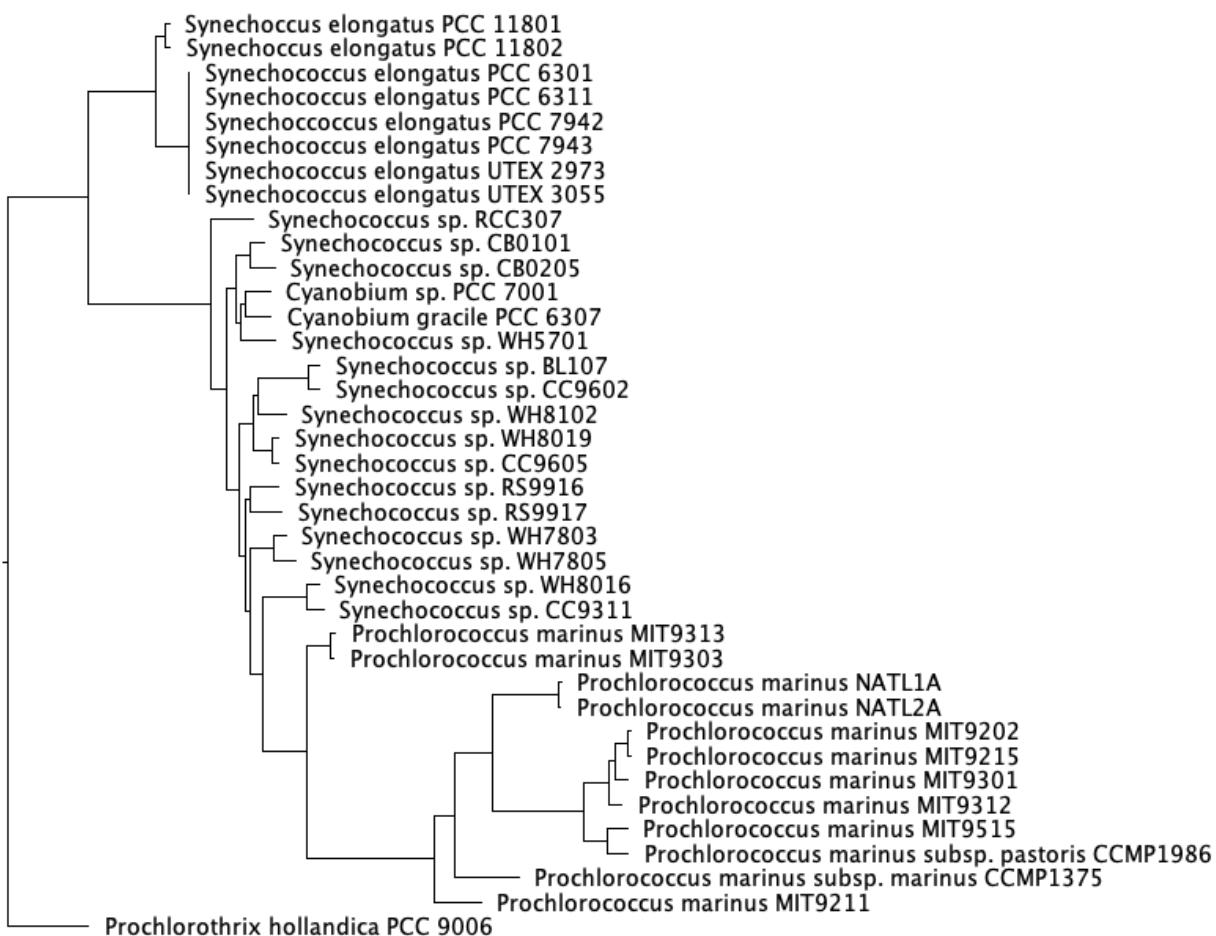

Tree scale = 0.2

Supplement: FIG S1 [file mbio.00862-22-s0009.pdf]

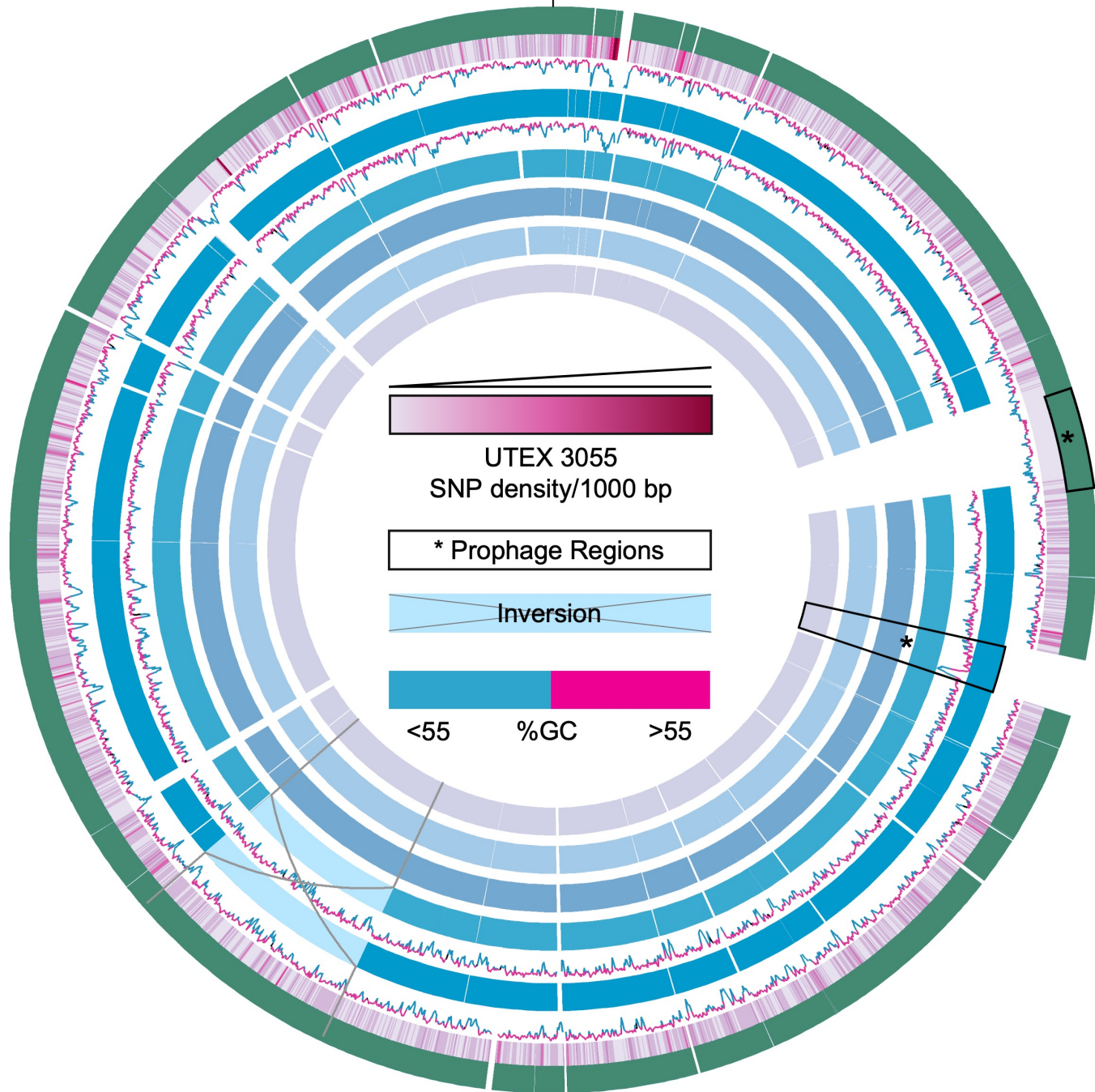

Supplement: FIG S2 [file mbio.00862-22-s0008.pdf]

# UTEX 3055 Unique Gene Set

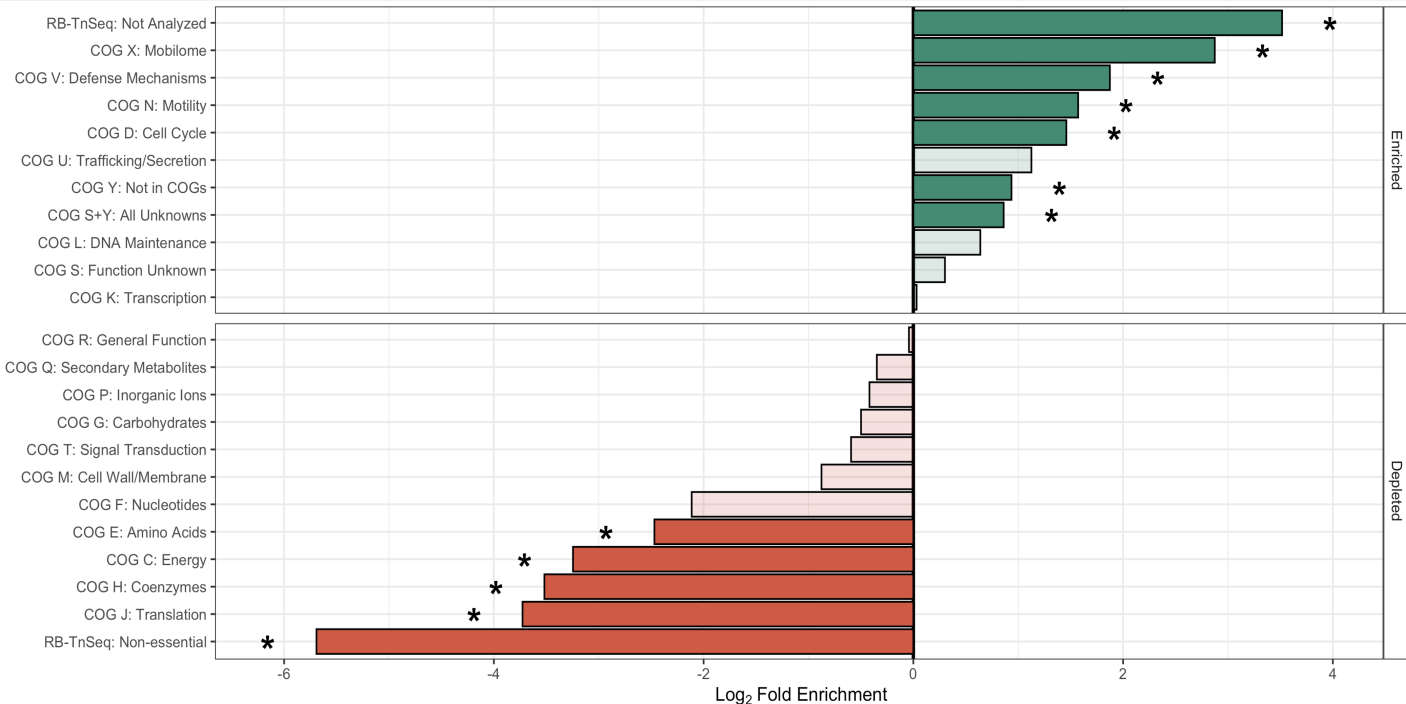

Supplement: FIG S4 [file mbio.00862-22-s0006.pdf]

# Disruption Cosmid

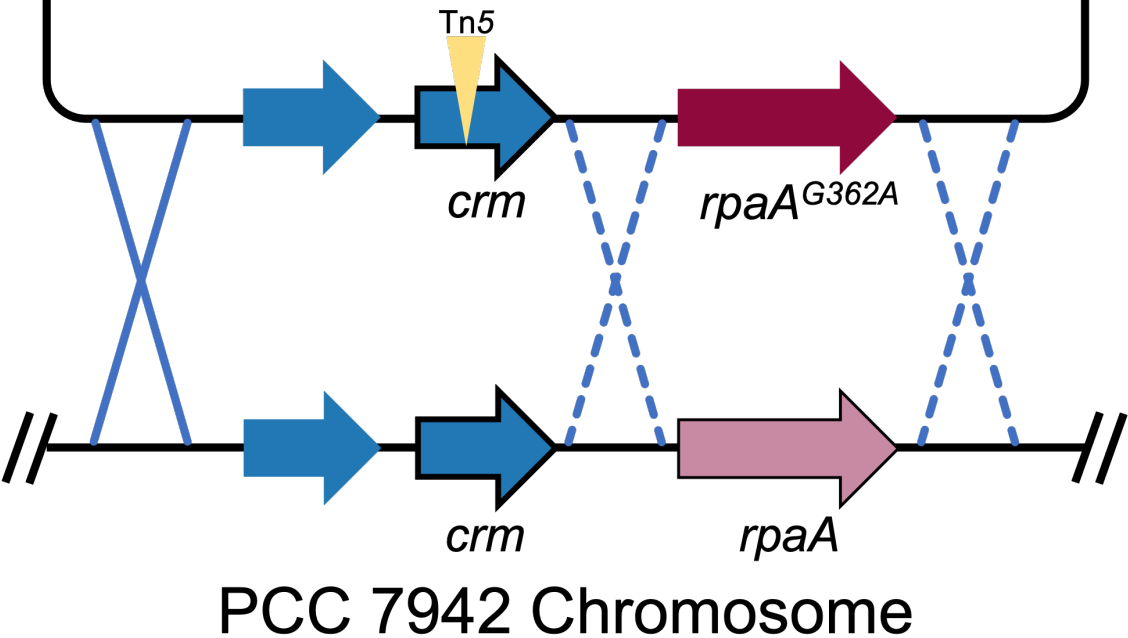

Supplement: FIG S5 [file mbio.00862-22-s0005.pdf]

A

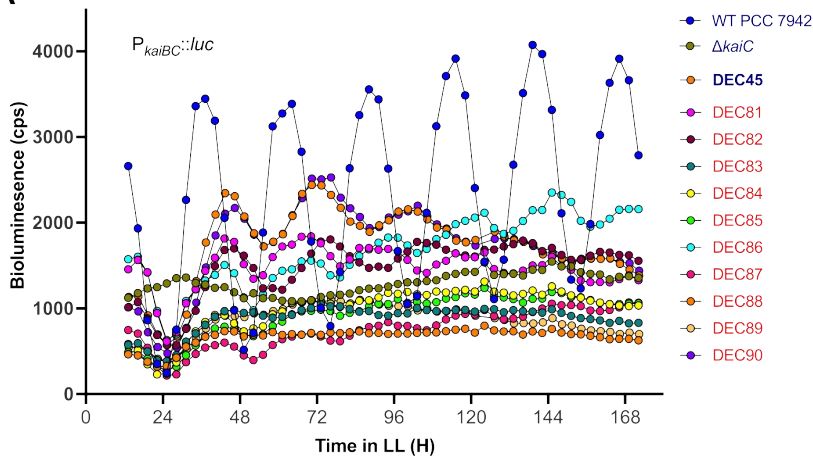

B

WT PCC 7942

 $\Delta kaiC$  $\Delta rpaA$ 

DEC81

DEC82

DEC83

DEC84

DEC85

DEC86

DEC87

DEC88

DEC89

DEC90

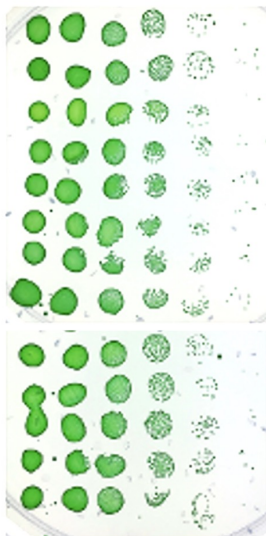

LL

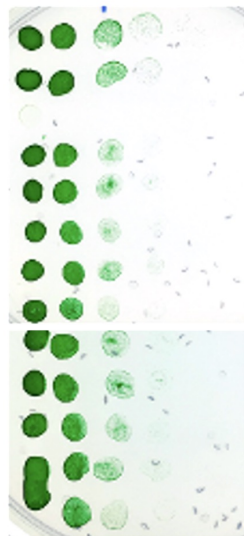

12:12 LD

Supplement: FIG S6 [file mbio.00862-22-s0004.pdf]

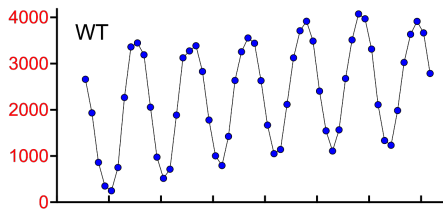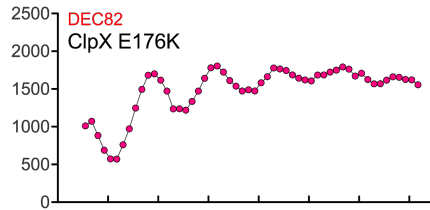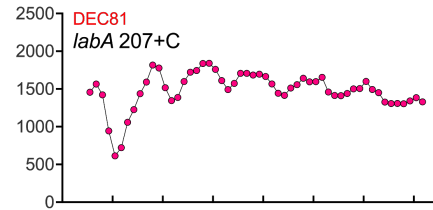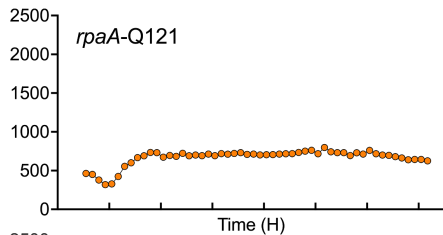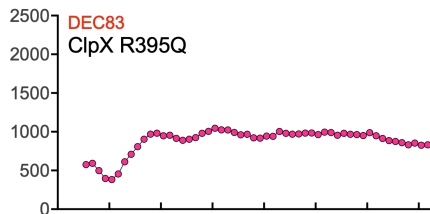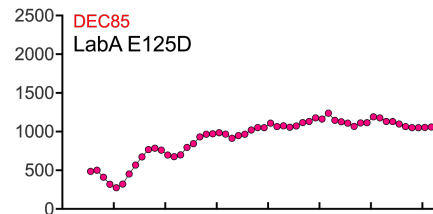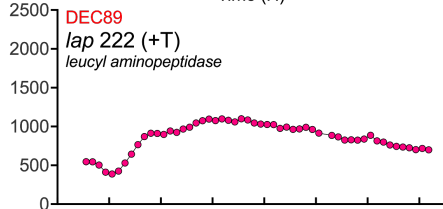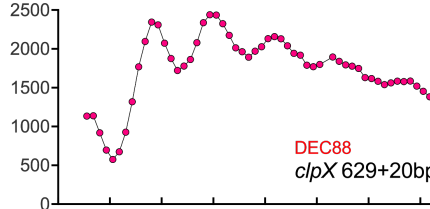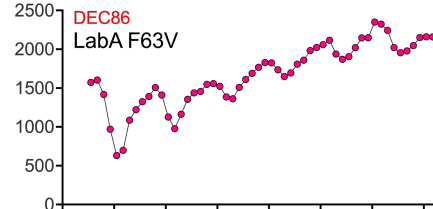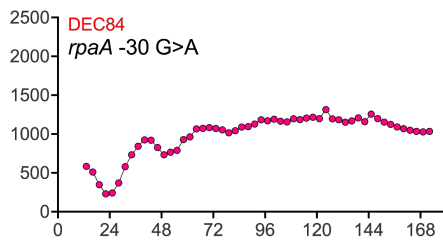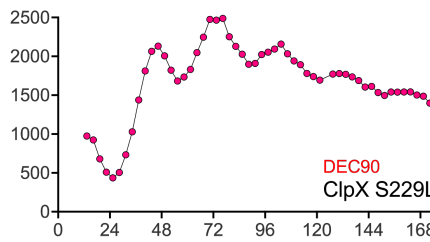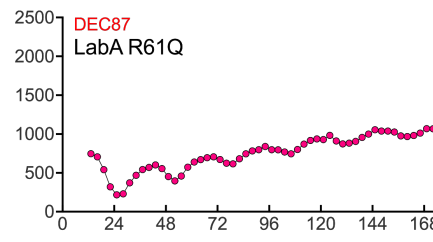

Supplement: FIG S7 [file mbio.00862-22-s0003.pdf]
