# Supplementary material for: Comparative Genomics of Synechococcus elongatus Explains the Phenotypic Diversity of the Strains
Source: mBio. 2022 Apr 27;13(3):e00862-22. doi: 10.1128/mbio.00862-22 (PMC9239245; doi:10.1128/mbio.00862-22)
Supplement: FIG S3 [file mbio.00862-22-s0007.pdf]

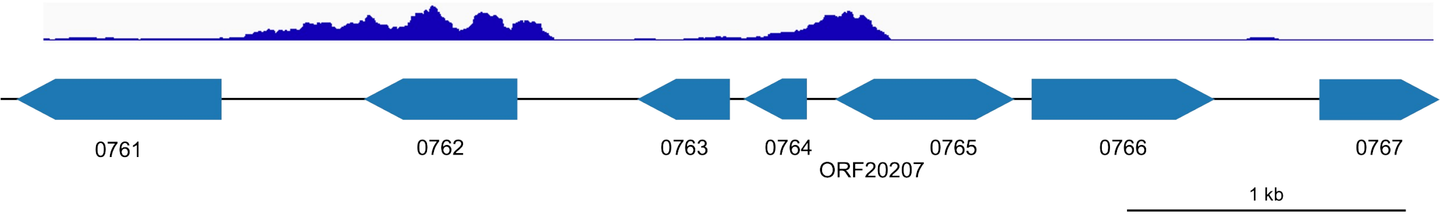


| Locus | Description |
| --- | --- |
| Synpcc7942_0761 | conserved hypothetical protein DUF2971 |
| Synpcc7942_0762 | DUF4065 phage-associated HTH antitoxin XRE family |
| Synpcc7942_0763 | XRE family antitoxin, phage immunity repressor protein C |
| Synpcc7942_0764 | XRE family antitoxin, phage immunity repressor protein C |
| ORF02027 | XRE family HTH antidote protein HigA, Cro/C1 type |
| Synpcc7942_0765 | tetraacyldisaccharide-1-P-4-kinase, tRNA methylation domain |
| Synpcc7942_0766 | Bro-N domain, phage antirepressor protein |
| Synpcc7942_0767 | transcription termination factor Rho |
